# ﻿Two new karst-adapted species in the *Cyrtodactyluspulchellus* group (Reptilia, Gekkonidae) from southern Thailand

**DOI:** 10.3897/zookeys.1179.109712

**Published:** 2023-09-14

**Authors:** Korkhwan Termprayoon, Attapol Rujirawan, L. Lee Grismer, Perry L. Wood Jr, Anchalee Aowphol

**Affiliations:** 1 Animal Systematics and Ecology Speciality Research Unit, Department of Zoology, Faculty of Science, Kasetsart University, Bangkok 10900, Thailand; 2 Biodiversity Center, Kasetsart University, Bangkok 10900, Thailand; 3 Herpetology Laboratory, Department of Biology, La Sierra University, 4500 Riverwalk Parkway, Riverside, California 92515, USA; 4 Department of Herpetology, San Diego Natural History Museum, PO Box 121390, San Diego, California, 92112, USA; 5 Institute for Tropical Biology and Conservation, Universiti Malaysia Sabah, Jalan UMS, 88400 Kota Kinabalu, Sabah, Malaysia; 6 Department of Ecology and Evolutionary Biology, University of Michigan, Ann Arbor, Michigan 48109, USA

**Keywords:** *Cyrtodactylussungaiupe* sp. nov., *Cyrtodactyluswangkhramensis* sp. nov., morphology, phylogeny, Southeast Asia, Sundaland, taxonomy, Thai-Malay Peninsula

## Abstract

The exploration of unsurveyed areas in southern Thailand discovered two new karst-adapted species, *Cyrtodactylussungaiupe***sp. nov.** and *Cyrtodactyluswangkhramensis***sp. nov.**, from Thung Wa and La-ngu Districts, Satun Province, respectively. These new species are members of the *C.pulchellus* group that occur along the Thai-Malay Peninsula. The new species can be distinguished from all other congeners by their key morphological characters and genetic divergence. Morphologically, *Cyrtodactylussungaiupe***sp. nov.** and *Cyrtodactyluswangkhramensis***sp. nov.** can be diagnosed from other members by having a combination of differences in body size; degree of dorsal tuberculation; absence of tubercles on ventral surfaces; number of ventral scales, paravertebral tubercles and femoroprecloacal pores in males only; deep precloacal groove only in males; absence of a scattered pattern of white dorsal tubercles; number of dark body bands; and the extent of caudal tubercles on an original tail. Although the two species are sister taxa and have nearly identical morphologies, they are considered to be different species, based on a relatively high uncorrected pairwise genetic divergence of the mitochondrial ND2 gene (6.59–6.89%), statistically significant univariate and multivariate morphological differences (PERMANOVA and ANOVA) and diagnostic characteristics of caudal tuberculation on the original tail. Moreover, *Cyrtodactylussungaiupe***sp. nov.** and *Cyrtodactyluswangkhramensis***sp. nov.** are currently restricted to their karstic type localities which may serve as a geographic barrier to dispersal and gene flow.

## ﻿Introduction

The bent-toed gecko genus *Cyrtodactylus* is the most diverse gekkotan group and is the third largest vertebrate genus in the world (Grismer et al. 2021a). This monophyletic genus contains 340 named species (Bohra et al. 2022; Grismer et al. 2022a; Uetz et al. 2022) within 32 well-supported monophyletic lineages (or species groups; Grismer et al. [2022b]). *Cyrtodactylus* is widely distributed across South Asia to Australia and western Melanesia, covering at least eight geographic regions (Grismer et al. 2020, 2021b, 2022b). However, most members have narrow geographic distributions (e.g. Pauwels et al. 2018; Chandramouli 2020; Nguyen et al. 2021; Termprayoon et al. 2021a). The diversity of *Cyrtodactylus* has evolved in geologically complex regions of Southeast Asian countries (e.g. Nguyen et al. 2021; Riyanto et al. 2022; Chan et al. 2023) including Thailand which currently contains 46 named species (Uetz et al. 2022; Grismer et al. 2023). The rate of newly-described species has been remarkable and has continued to increase in past decades owing to the exploration of unsurveyed areas coupled with integrative taxonomic studies, revealing the hidden species within previously-known species (e.g. Agarwal et al. 2018; Murdoch et al. 2019; Davis et al. 2020; Chomdej et al. 2021; Grismer et al. 2021c). Despite the high rate of new species discoveries of *Cyrtodactylus*, there are still poorly-known regions awaiting surveys that likely contain undescribed species awaiting formal descriptions (O’Connell et al. 2019; Chomdej et al. 2021; Grismer et al. 2023).

*Cyrtodactyluspulchellus* Gray, 1827 was considered a single widespread species distributed along the southern Thai-Malay Peninsula to southern Peninsular Malaysia and thought to be a single species for nearly two centuries (Grismer et al. 2016). Grismer (2011) suggested that the variation within these species may indicate that it is a species complex. Subsequently, an integrative approach was used to recover several morphologically and genetically distinct species within this *C.pulchellussensu lato* (Grismer et al. 2012, 2014a, 2016; Quah et al. 2019; Wood et al. 2020). Based on molecular data, the *C.pulchellus* species group is monophyletic and currently comprises 17 named species (Grismer et al. 2012, 2014a, 2016; Quah et al. 2019; Wood et al. 2020; Termprayoon et al. 2021a, b), of which four occur in Thailand: *C.astrum* Grismer, Wood, Quah, Anuar, Muin, Sumontha, Ahmad, Bauer, Wangkulangkul, Grismer & Pauwels, 2012, *C.lekaguli* Grismer, Wood, Quah, Anuar, Muin, Sumontha, Ahmad, Bauer, Wangkulangkul, Grismer & Pauwels, 2012, *C.macrotuberculatus* Grismer & Ahmad, 2008 and *C.stellatus* Termprayoon, Rujirawan, Ampai, Wood & Aowphol, 2021. Members of this species group occupy a variety of habitats which can be categorised as general, granite and karst (Grismer et al. 2021b). In the last few years, several new species were discovered from karst habitats (Quah et al. 2019; Wood et al. 2020; Termprayoon et al. 2021a) within the known range of the species group and it has been hypothesised that additional species from unexplored karstic landscape will be found.

During a herpetological survey in Satun Province, southern Thailand, two populations (Thung Wa and La-ngu Districts) of the *C.pulchellus* group were collected from karst formations. Molecular and morphological studies of these two populations revealed that they differ from currently recognised species and from each other. Herein, the two populations are considered to be new species and are described below.

## ﻿Materials and methods

### ﻿Sampling

**Figure 1.**
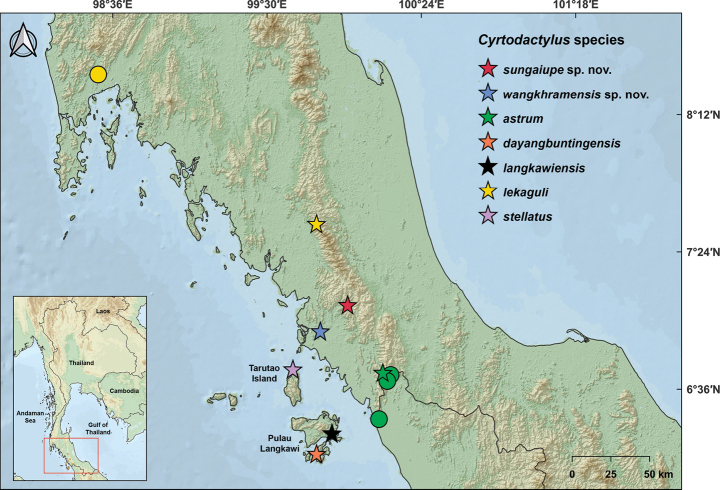
Map showing the type localities of *Cyrtodactylussungaiupe* sp. nov. (red star) in Thung Wa District and *Cyrtodactyluswangkhramensis* sp. nov. (blue star) in La-ngu District, Satun Province, Thailand and the type localities of closely-related species, *C.astrum*, *C.dayangbuntingensis*, *C.langkawiensis*, *C.lekaguli* and *C.stellatus*. Circles represent additional localities of specimens used in molecular analyses.

Field surveys were conducted in karst formations in Thung Wa and La-ngu Districts, Satun Province, southern Thailand (Fig. 1; Suppl. material 1). The specimens of the *C.pulchellus* group were generally observed at night (1900–2100 h) between October 2016 to April 2022. Geographical coordinates and elevations of each locality were recorded using a Garmin GPSMAP 64s. Ambient temperature and relative humidity were recorded using a Kestrel 400 Weather Meter. Ecological data at the time of capture (e.g. time, microhabitat and substrate) were recorded for each specimen. Specimens were euthanised by injecting tricaine methanesulphonate (MS-222). Liver tissue was removed and preserved in 95% ethyl alcohol and stored at -20 °C for genetic study. Prior to permanent storage in 70% ethyl alcohol, voucher specimens were initially fixed in 10% formalin for morphological study. Voucher specimens were deposited in the herpetological collections of the Zoological Museum, Kasetsart University, Thailand (ZMKU).

### ﻿Molecular analyses

Total genomic DNA of 14 newly-collected specimens was extracted from ethanol-preserved liver tissues using a NucleoSpin Tissue Kit (Macherey-Nagel GmbH & Co. KG, Germany) with standard manufacturer’s protocols. A partial sequence of the mitochondrial NADH dehydrogenase subunit 2 (ND2) gene and flanking tRNAs was amplified using polymerase chain reaction (PCR) under the following conditions: initial denaturation at 94 °C for 4 min, followed by 33–35 cycles of denaturation at 94 °C for 30 sec, annealing at 48–52 °C for 30 sec, extension at 72 °C for 90 sec and final extension at 72 °C for 7 min using the Metf6 (5’-AAGCTTTCGGGCCCATACC-3’) and COIH (5’-AGRGTGCCAATGTCTTTGTGRTT-3’) primer pairs, following Macey et al. (1997). All PCR products were purified and sequenced for forward and reverse strands using amplifying primers on ABI 3730XL DNA Sequencer by Sangon Biotech Inc. (Shanghai, China) using BigDye version 3 chemistry (Applied Biosystems, CA, USA). Sequences were edited and manually checked in Geneious R11 (Biomatters Ltd., Auckland, New Zealand). All newly-generated sequences were deposited in GenBank under the accession numbers OR346980–OR346993 (Suppl. material 1).

Homologous sequences of other species in the *C.pulchellus* group and seven outgroup species *Agamurapersica* (Duméril, 1856), *C.elok* Dring, 1979, *C.hontreensis* Ngo, Grismer & Grismer, 2018, *C.interdigitalis* Ulber, 1993, *C.intermedius* (Smith, 1917), *Hemidactylusfrenatus* Duméril & Bibron, 1836 and *Tropiocolotessteudneri* (Peters, 1869) were downloaded from GenBank. Outgroup species used to root the tree were based on Wood et al. (2012). Newly-generated sequences were aligned to all downloaded sequences using the MUSCLE plug-in implemented in Geneious R11. The protein-coding region of ND2 was translated to amino acid and checked for erroneous stop codons. All sequences used are listed in Suppl. material 1.

Maximum Likelihood (ML) and Bayesian Inference (BI) analyses were used to estimate phylogenetic trees. The ND2 dataset was partitioned by codon position for the protein-coding region and the tRNAs were treated as a separate partition. The evolutionary model selected for each partition was determined using ModelFinder with the Bayesian Information Criterion (BIC) as implemented in IQ-TREE (Kalyaanamoorthy et al. 2017). The selected models for ML and BI are listed in Table 1. The ML analysis was performed in the IQ-TREE webserver (Trifinopoulos et al. 2016). The ultrafast bootstrap approximation (UFB; Hoang et al. [2018]) was used to construct a final consensus tree via 1,000 bootstrap replicates. Nodes with UFB values of ≥ 95 were considered to be strongly supported (Minh et al. 2013). The BI analysis was run with MrBayes 3.2.6 on XSEDE (Ronquist et al. 2012) implemented in the CIPRES Science Gateway v.3.3 (Miller et al. 2010) with the models of evolution most closely approximating those calculated for the ML analysis. Two independent runs were performed with four chains per run, three hot and one cold. The Markov Chain Monte Carlo (MCMC) chains were run for 10 million generations and sampled every 1,000 generations, with 25% of each run discarded as burn-in. Stationarity and the effective sample sizes (ESS) for all parameters were assessed in Tracer v. 1.7.1. (Rambaut et al. 2018). Nodes with Bayesian posterior probabilities of ≥ 0.95 were considered strongly supported (Huelsenbeck and Ronquist 2001; Wilcox et al. 2002). Uncorrected pairwise genetic divergences (*p*-distance) were calculated for both intra- and interspecific using the default settings in MEGA 11 (Tamura et al. 2021).

**Table 1.** The evolutionary model selected of partitioning ND2 gene and tRNAs estimated by BIC implemented in IQ-TREE. Selected models were applied for the Maximum Likelihood (ML) and Bayesian Inference (BI) analyses.

Gene	Model selected	Model applied for ML	Model applied for BI
**ND2**			
1^st^ position	TN+F+G4	TN+F+G4	GTR+I+Γ
2^nd^ position	TIM3+F+G4	TIM3+F+G4	GTR+I+Γ
3^rd^ position	TIM2+F+I+G4	TIM2+F+I+G4	GTR+I+Γ
**tRNAs**	TN+F+G4	TN+F+G4	GTR+I+Γ

### ﻿Morphology

The morphological examination was conducted on preserved specimens of the Thung Wa population, the La-ngu population, *C.astrum*, *C.lekaguli* and *C.stellatus* (Appendix 1). For *C.lekaguli*, only type series and specimens from the type locality (FMNH, THNHM and ZMKU) were examined. The institutional abbreviations of examined specimens follow Sabaj (2020). All specimens were examined by the first author, except type specimens of *C.lekaguli*, which was examined by Attapol Rujirawan. Characters and abbreviations were modified from previous studies of the *C.pulchellus* group (Grismer et al. 2016; Quah et al. 2019; Wood et al. 2020; Termprayoon et al. 2021a), the *C.peguensis* group (Grismer et al. 2018) and the *C.oldhami* group (Grismer et al. 2018; Yodthong et al. 2022). Morphometrics were measured using digital calipers to the nearest 0.1 mm on the left side of the body. Sixteen measurements were as follows:

**SVL** Snout-vent length, taken from the tip of snout to the vent;

**TW** Tail width, taken at the base of the tail immediately posterior to the postcloacal swelling;

**TL** Tail length, taken from vent to the tip of the tail, original or regenerated;

**FL** Forearm length, taken from the posterior margin of the elbow while flexed 90° to the inflection of the flexed wrist;

**TBL** Tibia length, taken from the posterior surface of the knee while flexed 90° to the base of the heel;

**AG** Axilla to groin length, taken from the posterior margin of the forelimb at its insertion point on the body to the anterior margin of the hind limb at its insertion point on the body;

**HL** Head length, the distance from the posterior margin of the retroarticular process of the lower jaw to the tip of the snout;

**HW** Head width, measured at the angle of the jaws;

**HD** Head depth, the maximum height of head from the occiput to the throat;

**ED** Eye diameter, the greatest horizontal diameter of the eye-ball;

**EE** Eye to ear distance, measured from the anterior edge of the ear opening to the posterior edge of the eye-ball;

**ES** Eye to snout distance, measured from anterior-most margin of the eye-ball to the tip of snout;

**EN** Eye to nostril distance, measured from the anterior margin of the eye-ball to the posterior margin of the external nares;

**IO** Inter orbital distance, measured between the anterior edges of the orbit;

**EL** Ear length, the greatest vertical distance of the ear opening;

**IN** Internarial distance, measured between the nares across the rostrum.

Meristic characters were evaluated under a Nikon SMZ745 dissecting microscope on both left (L) and right (R) sides, when possible, for the following eighteen characters:

**SL** Supralabial scales, counted from the largest scale immediately posterior to the dorsal inflection of the posterior portion of the upper jaw to the rostral scale;

**SL-mid-eye** The numbers of supralabial scales, counted from the largest scale immediately below the middle of the eye-ball to the rostral scales;

**IL** Infralabial scales, counted from the largest scale immediately posterior to the dorsal inflection of the posterior portion of the upper jaw to the mental scale;

**IL-mid-eye** The numbers of infralabial scales, counted from the largest scale immediately below the middle of the eye-ball to the mental scales;

**PVT** The number of paravertebral tubercles between limb insertions, counted in a straight line immediately left or right of the vertebral column;

**LRT** The number of longitudinal rows of body tubercles, counted transversely across the centre of the dorsum from one ventrolateral fold to the other;

**VS** The number of longitudinal rows of ventral scales, counted transversely across the centre of the abdomen from one ventrolateral fold to the other;

**4FLU** The number of small, unmodified subdigital lamellae distal to the digital inflection on the fourth finger, counted from the digital inflection to the claw;

**4FLE** The number of expanded subdigital lamellae proximal to the digital inflection on the fourth finger, counted from the base of the first phalanx where it contacts the body of the hand to the largest scale on the digital inflection;

**4FL** The total number of subdigital lamellae beneath the fourth finger;

**4TLU** The number of small, unmodified subdigital lamellae distal to the digital inflection on the fourth toe, counted from the digital inflection to the claw;

**4TLE** The number of expanded subdigital lamellae proximal to the digital inflection on the fourth toe, counted from the base of the first phalanx where it contacts the body of the foot to the largest scale on the digital inflection;

**4TL** The total number of subdigital lamellae beneath the fourth toe, counted from the base of the first phalanx to the claw;

**FPP** The total number of precloacal and femoral pores in male (i.e. the sum of the number of femoral and precloacal scales bearing pores combined as a single meristic referred to as the femoroprecloacal pores);

**PCT** The number of rows and total number of postcloacal (hemipenial) tubercles in adult male;

**BB** The number of dark body bands between limb insertions;

**LCB** The number of light caudal bands on the original tail;

**DCB** The number of dark caudal bands on the original tail.

Additional non-meristic characters evaluated were the degree of body tuberculation, weak tuberculation refers to dorsal body tubercles that are low and rounded, whereas prominent tuberculation refers to tubercles that are raise and keeled; the presence or absence of tubercles on the dorsal and ventral surfaces of the forearms; the presence or absence of tubercles in the gular region, throat and ventrolateral body folds; body tubercles extending past the base of the tail or not; the width of the dark body bands relative to the width of the interspace between the bands; the presence or absence of dark pigmentation infused in the white caudal bands of adults; the presence of caudal tubercles; the presence or absence of a precloacal depression or groove; femoroprecloacal pore continuous or not; the presence or absence of scattered white/yellow tubercles on the dorsum; and the presence or absence of white tail tip on the posterior portion of the original tail in hatchlings and juveniles. Colour pattern was taken on dorsal, ventral, lateral image of the body in both sexes and of all possible age classes prior to preservation.

Additional morphological data for analyses and comparisons were obtained from the original descriptions of other species in the *C.pulchellus* group (Grismer et al. 2012; Quah et al. 2019; Wood et al. 2020; Termprayoon et al. 2021a).

### ﻿Statistical analyses

**Figure 2.**
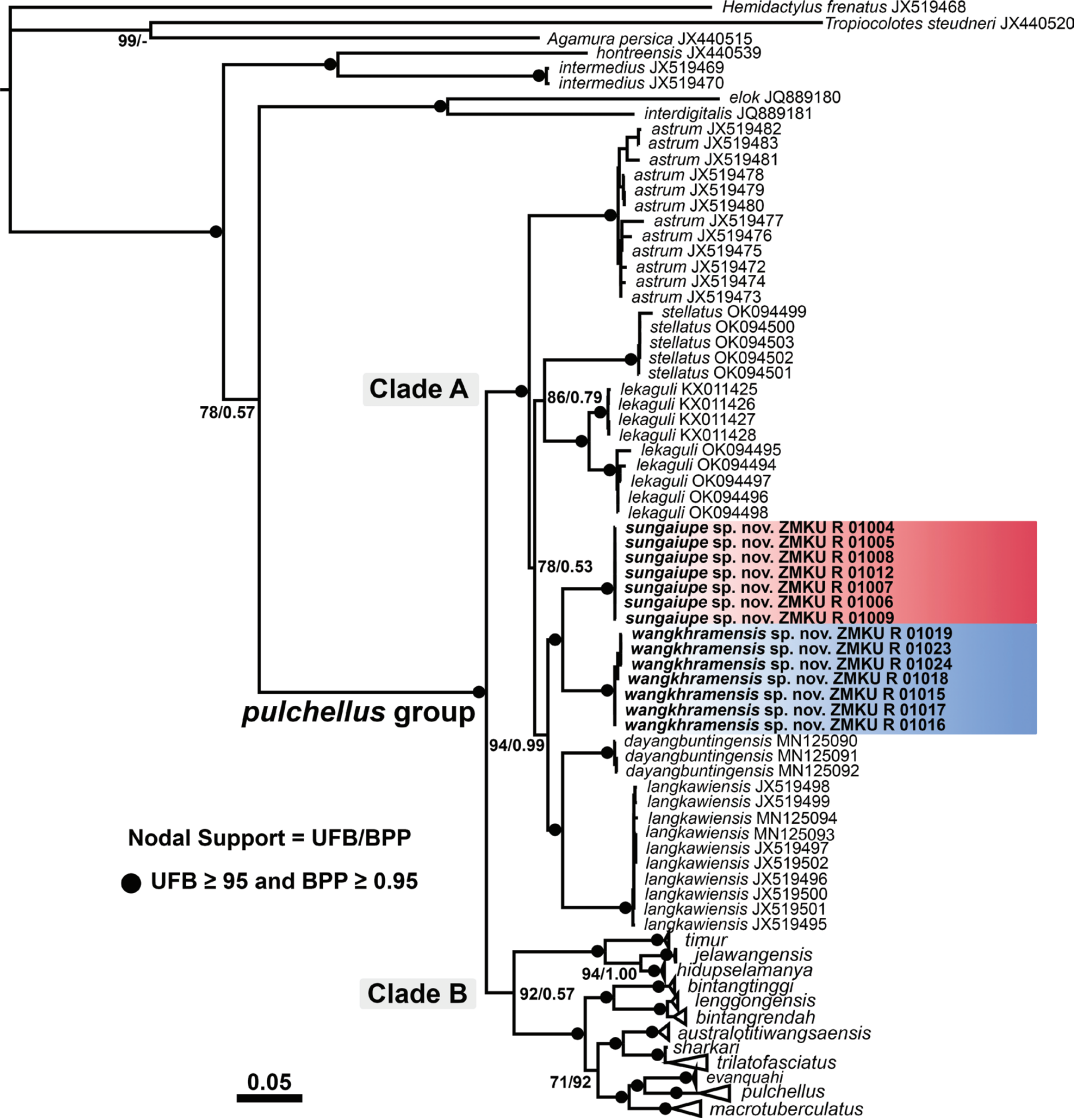
Maximum Likelihood phylogenetic tree of *Cyrtodactylussungaiupe* sp. nov. and *Cyrtodactyluswangkhramensis* sp. nov. within the *C.pulchellus* group reconstructed from 1,444 bp of ND2 and flanking tRNAs. Nodes show ultrafast bootstrap support (UFB) and Bayesian posterior probabilities (BPP) values.

Based on the phylogenetic tree, morphological analyses were performed on seven lineages (= species) in Clade A (Fig. 2) including the Thung Wa population (*n* = 8), the La-ngu population (*n* = 12), *C.astrum* (*n* = 8), *C.dayangbuntingensis* (*n* = 2), *C.langkawiensis* (*n* = 6), *C.lekaguli* (*n* = 26), and *C.stellatus* (*n* = 10).

Statistical analyses were conducted using the software R (R Core Team 2019). Tail characters (TL and TW) were excluded due to their different conditions (e.g. original, regenerated or broken). Morphometric characters (except SVL) were size-adjusted in order to eliminate bias of allometry by allometric equation: X_adj_ = log[X ± β(SVL ± SVL_mean_)], where X_adj_ = adjusted value; X = measured value; β = unstandardised regression coefficient for each species; SVL = measured snout-vent length; SVL_mean_ = overall average SVL of each allometry species (Thorpe 1975, 1983; Turan 1999; Lleonart et al. 2000)—implemented through the R package GroupStruct (Chan and Grismer 2021). The adjustments are automatically conducted separately on each species and then concatenated into a single data frame to ensure there is no interspecific conflation of variation (Reist 1985; McCoy et al. 2006).

Fourteen morphometrics (SVL, FL_adj_, TBL_adj_, AG_adj_, HL_adj_, HW_adj_, HD_adj_, ED_adj_, EE_adj_, ES_adj_, EN_adj_, IO_adj_, EL_adj_ and IN_adj_) and seven meristics (SL, IL, PVT, LRT, VS, 4TL and BB) were concatenated to a single dataset. Femoroprecloacal pore counts were excluded from analyses due to their presence in only males. Some meristic characters (SL-mid-eye, IL-mid-eye, 4FLU, 4FLE, 4FL, 4TLU and 4TLE) were omitted due to inadequate data of some species. The dataset was analysed using principal components analysis (PCA) to reduce noise in the dataset and explore interspecific differences in morphospace amongst species using the FactorMineR package (Lê et al. 2008). The first two PC plots were visualised using ggplot2 package (Wickham 2016). The significant differences in centroid locations and group clustering amongst species were determined using a non-parametric permutation-multivariate analysis of variance (PERMANOVA) from the *vegan* package in R (Oksanen et al. 2020). A Euclidean (dis)similarity matrix using 50,000 permutations was used in the analysis, based on the loadings of the first four dimensions recovered from the PCA. The PCAtest (Camargo 2022) was used to evaluate the overall significance of the PCA dataset, the significance of the PC axes, based on their eigenvalues (i.e. determining which axes contained statistically significant signal) and the contribution of each of the observed variables of the significant axes across the dataset. This removes the partially subjective and *ad hoc* thresholds used to describe non-trivial PC axes. The function runs 1,000 random permutations and bootstrap replicates of the empirical data. Based on the bootstrap resampling and permutation, 95%-confidence intervals around mean values were calculated. Significant *p*-values imply there is non-random correlational structure in the overall dataset and that the PCA is biologically meaningful. Statistically-significant eigenvalues indicate their respective PC axes reflect non-random correlations amongst variables and statistically significant loadings indicate their respective variables have a larger contribution in the PC score beyond random noise. This test delivers statistically defensible quantitative results by delimiting which PC axes contain signal and opposed to noise and, in so doing, illuminates the most significant PC axes influencing the results of the PERMANOVA.

For statistical comparison, the small sample size of *C.dayangbuntingensis* (*n* = 2) was excluded from the univariate analysis. Data of each species were tested for normality using the Shapiro-Wilk test (*p* ≥ 0.05). Normally distributed data were tested for homogeneity of variances using Levene’s test (*p* ≥ 0.05). Differences amongst species were compared using Analysis of Variance (ANOVA) and Tukey *post hoc* test (Tukey’s test) for normalised and equal variance data or Welch’s *F*-test and Games-Howell *post hoc* test for unequal variance data. Non-normalised characters were compared using non-parametric Kruskal-Wallis test and followed by a *post hoc* Dunn’s multiple comparison (Dunn’s test).

## ﻿Results

### ﻿Phylogenetic relationships

The aligned dataset of the partial ND2 gene and flanking tRNAs contained 1,444 bp of 100 individuals. The Maximum Likelihood value of the best ML tree was lnL = -15,495.881. The standard deviation of split frequencies was 0.002877 between the two simultaneous BI runs and the ESS values were ≥ 7594.3 for all parameters. The results of ML and BI phylogenetic analyses revealed two largely concordant topologies across the major nodes, with minor differences in support values. Only the ML tree is represented in this study (Fig. 2). Both analyses recovered the Thung Wa and La-ngu populations as members of the *C.pulchellus* group and each was embedded within Clade A, comprised of *C.astrum*, *C.dayangbuntingensis*, *C.langkawiensis*, *C.lekaguli* and *C.stellatus*. Each population formed a well-supported monophyletic lineage (100 UFB, 1.00 BPP), referred to Thung Wa lineage (*Cyrtodactylussungaiupe* sp. nov. [see below]) and La-ngu lineage (*Cyrtodactyluswangkhramensis* sp. nov. [see below]). These two lineages were strongly supported (98 UFB, 1.00 BPP) as sister taxa.

The intra- and interspecific sequence divergences of two new populations and other species in Clade A are shown in Table 2. The *p*-distances within *Cyrtodactylussungaiupe* sp. nov. were low, ranging from 0.00–0.07% (mean 0.02%) and range from 0.00–0.52% (mean 0.28%) within *Cyrtodactyluswangkhramensis* sp. nov. *Cyrtodactylussungaiupe* sp. nov. had uncorrected *p*-distance of 6.59–6.89% (mean 6.76%) from its sister species *Cyrtodactyluswangkhramensis* sp. nov. *Cyrtodactylussungaiupe* sp. nov. and *Cyrtodactyluswangkhramensis* sp. nov. had uncorrected *p*-distance of 8.02–10.69% and 7.57–11.30%, respectively, from the remaining species in Clade A (Table 2).

**Table 2.** Percentage uncorrected pairwise sequence divergences (*p*-distances) of *Cyrtodactylussungaiupe* sp. nov., *Cyrtodactyluswangkhramensis* sp. nov. and closely-related species (Clade A), based on 1,444 base pairs of mitochondrial ND2 gene and flanking tRNAs. The *p*-distance values are given as mean and ranges in parentheses. Intraspecific distances are in bold font.

Species	*n*	*Cyrtodactylussungaiupe* sp. nov.	*Cyrtodactyluswangkhramensis* sp. nov.	*C.astrum*	*C.dayangbuntingensis*	*C.langkawiensis*	*C.lekaguli*	*C.stellatus*
*Cyrtodactylussungaiupe* sp. nov.	7	**0.02 (0.00–0.07)**						
*Cyrtodactyluswangkhramensis* sp. nov.	7	6.76 (6.59–6.89)	**0.28 (0.00–0.52)**					
*C.astrum*	12	9.75 (9.18–10.69)	9.63 (8.97–11.01)	**1.44 (0.00–3.12)**				
*C.dayangbuntingensis*	3	8.10 (8.02–8.16)	7.84 (7.57–8.16)	9.56 (9.18–10.90)	**0.14 (0.07–0.21)**			
*C.langkawiensis*	10	8.86 (8.67–9.03)	8.46 (8.11–8.74)	10.06 (9.53–11.54)	7.38 (7.17–7.66)	**0.28 (0.00–0.52)**		
*C.lekaguli*	9	8.57 (8.11–9.23)	8.93 (8.16–9.52)	9.74 (8.99–11.59)	8.56 (8.00–9.20)	9.30 (8.42–10.08)	**2.20 (0.00–4.27)**	
*C.stellatus*	5	9.90 (9.59–10.47)	10.16 (9.62–11.30)	10.54 (9.85–12.34)	9.48 (9.21–10.29)	10.39 (9.98–11.37)	9.22 (8.35–10.60)	**0.42 (0.00–0.95)**

### ﻿Morphology

**Figure 3.**
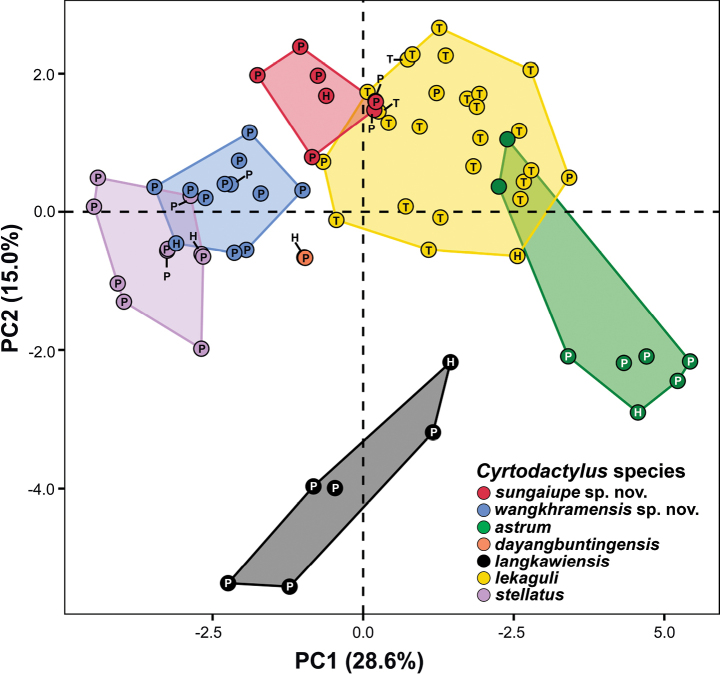
Principal Component Analysis (PCA) of *Cyrtodactylussungaiupe* sp. nov., *Cyrtodactyluswangkhramensis* sp. nov. and the closely-related species in Clade A of the *C.pulchellus* group. The letters in the scatter plots refer to holotype (= H), paratype (= P) and topotype (= T).

**Figure 4.**
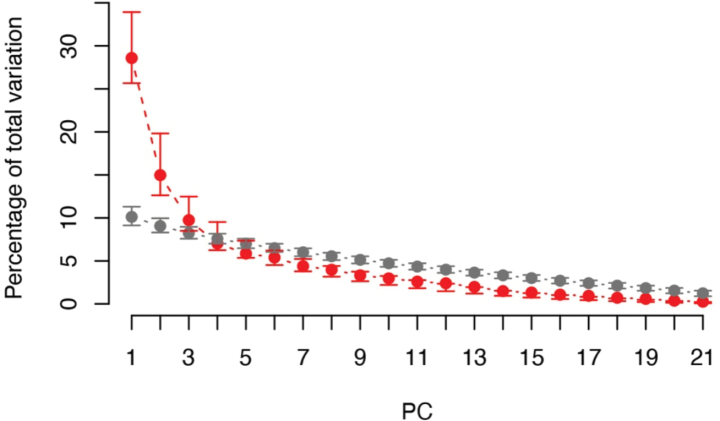
Statistically significant variation of the first three PCs based the permutation (grey) and bootstrap (red) analyses conducted in PCAtest.

The PCA analysis of the seven lineages in Clade A recovered morphological differences along the ordination of the first two PC axes (Fig. 3). The PC plot showed that *Cyrtodactylussungaiupe* sp. nov. clustered separately from *C.astrum*, *C.dayangbuntingensis*, *C.langkawiensis* and *Cyrtodactyluswangkhramensis* sp. nov. and only slightly overlapped with *C.lekaguli*. *Cyrtodactyluswangkhramensis* sp. nov. also clustered separately from all other species, except *C.stellatus* (partially overlapped). The first three components with eigenvalues > 1.00 (PC1–PC3) accounted for 53.30% of all variables and mostly loaded on morphometric characters (Table 3). PC1 explained 28.59% of the variation and was heavily loaded on FL_adj_, TBL_adj_, HW_adj_, HD_adj_, EE_adj_, ES_adj_ and EN_adj_. PC2 accounted for 14.97% of the variation and was heavily loaded on AG_adj_ and IN_adj_. PC3 accounted for 9.74% of the variation and was heavily loaded on EL_adj_. The PERMANOVA and PCAtest analyses indicated that the PCA contained a highly significant diagnostic signal amongst the lineages. PERMANOVA recovered the morphological clustering and centroid placement of *Cyrtodactylussungaiupe* sp. nov. and *Cyrtodactyluswangkhramensis* sp. nov. as significantly different (*p* < 0.001–0.036; Table 4) from each other and all other species in Clade A. PCAtest recovered PC1–3 as containing statistically different signal from the other 18 axes (*p* = 0.000 for all axes). Along PC1, all characters, except AG_adj_, EL_adj_, IN_adj_ and IL had statistically significant different loadings as opposed to the loadings of those same characters on the other axes (Fig. 4; Table 3). Statistically significant loadings for characters AG_adj_, IO_adj_, IN_adj_, PVT and VS were found along PC2 and along PC3, SL, PVT and LRT were statistically different from their respective loads on the other PC axes (Fig. 4; Table 3).

**Table 3.** Summary statistics and factor loadings of the first three principal components (PC1–3) of morphometric and meristic characters of *Cyrtodactylussungaiupe* sp. nov., *Cyrtodactyluswangkhramensis* sp. nov. and closely-related species including *C.astrum*, *C.dayangbuntingensis*, *C.langkawiensis*, *C.lekaguli* and *C.stellatus*. Bold fonts indicate high loadings. Abbreviations are listed in Materials and methods.

Characters	PC1	PC2	PC3
SVL	0.517	0.268	-0.160
FL_adj_	**0.692**	0.043	0.118
TBL_adj_	**0.696**	-0.048	0.372
AG_adj_	-0.017	**0.828**	-0.176
HL_adj_	0.475	0.255	0.112
HW_adj_	**0.880**	0.278	-0.008
HD_adj_	**0.794**	0.362	-0.157
ED_adj_	0.362	0.247	0.138
EE_adj_	**0.843**	0.162	-0.136
ES_adj_	**0.873**	-0.265	0.195
EN_adj_	**0.797**	-0.283	0.349
IO_adj_	0.431	0.433	-0.408
EL_adj_	0.002	-0.288	**0.725**
IN_adj_	-0.031	**0.798**	0.321
SL	-0.360	0.379	0.584
IL	-0.271	0.394	0.593
PVT	0.386	-0.507	0.003
LRT	0.505	-0.367	-0.181
VS	0.267	-0.471	0.141
4TL	0.108	0.137	0.009
BB	-0.162	-0.183	-0.248
Eigenvalue	6.003	3.144	2.046
Percentage of variance	28.587	14.971	9.743
Cumulative proportion	28.587	43.558	53.301

**Table 4.** Summary results of the PERMANOVA analysis of *Cyrtodactylussungaiupe* sp. nov., *Cyrtodactyluswangkhramensis* sp. nov. and closely-related species in Clade A. Bold fonts indicate significant differences.

Species	F model	R^2^	*p*-value	*p*-adjust
*Cyrtodactylussungaiupe* sp. nov. vs. *C.astrum*	36.943	0.725	**0.00018**	**0.00378**
*Cyrtodactylussungaiupe* sp. nov. vs. *C.dayangbuntingensis*	35.814	0.817	**0.02158**	0.45317
*Cyrtodactylussungaiupe* sp. nov. vs. *C.langkawiensis*	26.544	0.689	**0.00040**	**0.00840**
*Cyrtodactylussungaiupe* sp. nov. vs. *C.lekaguli*	18.448	0.366	**0.00002**	**0.00042**
*Cyrtodactylussungaiupe* sp. nov. vs. *C.stellatus*	37.059	0.698	**0.00004**	**0.00084**
*Cyrtodactyluswangkhramensis* sp. nov. vs. *Cyrtodactylussungaiupe* sp. nov.	23.125	0.562	**0.00004**	**0.00084**
*Cyrtodactyluswangkhramensis* sp. nov. vs. *C.astrum*	75.186	0.807	**0.00002**	0.**00042**
*Cyrtodactyluswangkhramensis* sp. nov. vs. *C.dayangbuntingensis*	40.165	0.770	**0.01102**	0.23142
*Cyrtodactyluswangkhramensis* sp. nov. vs. *C.langkawiensis*	22.629	0.586	**0.00008**	**0.00168**
*Cyrtodactyluswangkhramensis* sp. nov. vs. *C.lekaguli*	40.729	0.531	**0.00002**	**0.00042**
*Cyrtodactyluswangkhramensis* sp. nov. vs. *C.stellatus*	14.024	0.412	**0.00004**	**0.00084**
*C.astrum* vs. *C.dayangbuntingensis*	21.03	0.724	**0.02252**	0.47291
*C.astrum* vs. *C.langkawiensis*	15.765	0.568	**0.00016**	**0.00336**
*C.astrum* vs. *C.lekaguli*	29.859	0.483	**0.00002**	**0.00042**
*C.astrum* vs. *C.stellatus*	81.564	0.836	**0.00004**	**0.00084**
*C.dayangbuntingensis* vs. *C.langkawiensis*	7.269	0.548	**0.03571**	0.75000
*C.dayangbuntingensis* vs. *C.lekaguli*	13.583	0.343	**0.00264**	0.05544
*C.dayangbuntingensis* vs. *C.stellatus*	28.263	0.739	**0.01554**	0.32633
*C.langkawiensis* vs. *C.lekaguli*	28.755	0.489	**0.00002**	**0.00042**
*C.langkawiensis* vs. *C.stellatus*	20.318	0.592	**0.00020**	**0.00420**
*C.lekaguli* vs. *C.stellatus*	60.435	0.64	**0.00002**	**0.00042**

The significant differences recovered by the ANOVA (or Kruskal-Wallis test) amongst seven lineages in Clade A were found in most characters (*p* < 0.001–0.008), except 4TL and BB (*p* = 0.055–0.126) (Table 5; Suppl. material 2). The pairwise comparison post hoc tests showed that *Cyrtodactylussungaiupe* sp. nov. was significantly different from *Cyrtodactyluswangkhramensis* sp. nov. in four mensural characters of AG_adj_, HW_adj_, EE_adj_ and EN_adj_ (*p* < 0.001–0.009) and a meristic character of SL (*p* = 0.045). The mean values of significant characters were greater in the *Cyrtodactylussungaiupe* sp. nov. than *Cyrtodactyluswangkhramensis* sp. nov. (Table 5; Suppl. material 2). The summarised pairwise differences between *Cyrtodactylussungaiupe* sp. nov., *Cyrtodactyluswangkhramensis* sp. nov. and closely-related species in Clade A are shown in Table 5 and Suppl. material 2.

**Table 5.** Summary pairwise differences of statistically significant characters (Tukey’s test; *p* < 0.05) from morphometric and meristic characters of *Cyrtodactylussungaiupe* sp. nov., *Cyrtodactyluswangkhramensis* sp. nov. and closely-related species (Clade A). Abbreviations are listed in Materials and Methods. Key: * tested by Games-Howell test; ** tested by Dunn’s test. No significantly different characters of 4TL and BB were excluded.

**Characters**	SVL**	FL_adj_*	**TBL_adj_**	AG_adj_*	**HL_adj_**	**HW_adj_**	HD_adj_*	**EE_adj_**	**ED_adj_**	ES_adj_**
*Cyrtodactylussungaiupe* sp. nov. vs. *C.astrum*		< 0.001	< 0.001	0.006		< 0.001	0.016	0.004		0.008
*Cyrtodactylussungaiupe* sp. nov. vs. *C.langkawiensis*				0.001	< 0.001	0.033				
*Cyrtodactylussungaiupe* sp. nov. vs. *C.lekaguli*						< 0.001	0.001	0.008		
*Cyrtodactylussungaiupe* sp. nov. vs. *C.stellatus*	0.035	0.009	0.002	< 0.001	< 0.001	< 0.001	< 0.001	< 0.001		0.005
*Cyrtodactyluswangkhramensis* sp. nov. vs. *Cyrtodactylussungaiupe* sp. nov.				0.003		0.008		< 0.001		
*Cyrtodactyluswangkhramensis* sp. nov. vs. *C.astrum*	0.004	< 0.001	< 0.001		< 0.001	< 0.001	< 0.001	< 0.001		< 0.001
*Cyrtodactyluswangkhramensis* sp. nov. vs. *C.langkawiensis*				0.028	0.037					0.001
*Cyrtodactyluswangkhramensis* sp. nov. vs. *C.lekaguli*	< 0.001	0.007			< 0.001	< 0.001	< 0.001	< 0.001		< 0.001
*Cyrtodactyluswangkhramensis* sp. nov. vs. *C.stellatus*				0.048	0.015	< 0.001	0.014			
*C.astrum* vs. *C.langkawiensis*	0.031		< 0.001		< 0.001	< 0.001		0.004	< 0.001	
*C.astrum* vs. *C.lekaguli*		< 0.001	< 0.001	0.032						0.030
*C.astrum* vs. *C.stellatus*	0.002	< 0.001	< 0.001		< 0.001	< 0.001	< 0.001		< 0.001	< 0.001
*C.langkawiensis* vs. *C.lekaguli*	0.012			0.005	< 0.001	< 0.001		0.020	< 0.001	
*C.langkawiensis* vs. *C.stellatus*						< 0.001				< 0.001
*C.lekaguli* vs. *C.stellatus*	< 0.001	< 0.001	0.004	< 0.001	< 0.001	< 0.001	< 0.001		< 0.001	< 0.001
**Characters**	EN_adj_**	IO_adj_*	**EL_adj_**	IN_adj_**	SL**	IL**	PVT**	LRT**	VS*	
*Cyrtodactylussungaiupe* sp. nov. vs. *C.astrum*	0.043			0.009	0.004	0.011	< 0.001	< 0.001		
*Cyrtodactylussungaiupe* sp. nov. vs. *C.langkawiensis*				< 0.001	< 0.001	< 0.001	0.046	< 0.001	0.001	
*Cyrtodactylussungaiupe* sp. nov. vs. *C.lekaguli*		< 0.001	0.001		< 0.001	0.006		0.001		
*Cyrtodactylussungaiupe* sp. nov. vs. *C.stellatus*	< 0.001						0.003			
*Cyrtodactyluswangkhramensis* sp. nov. vs. *Cyrtodactylussungaiupe* sp. nov.	0.009				0.045					
*Cyrtodactyluswangkhramensis* sp. nov. vs. *C.astrum*	< 0.001		0.003				< 0.001	< 0.001		
*Cyrtodactyluswangkhramensis* sp. nov. vs. *C.langkawiensis*	0.012			0.010	0.010	0.002	0.005	< 0.001	< 0.001	
*Cyrtodactyluswangkhramensis* sp. nov. vs. *C.lekaguli*	0.002	< 0.001			0.036		0.002	0.001		
*Cyrtodactyluswangkhramensis* sp. nov. vs. *C.stellatus*							< 0.001			
*C.astrum* vs. *C.langkawiensis*										
*C.astrum* vs. *C.lekaguli*	0.007	< 0.001	< 0.001	0.018			0.002			
*C.astrum* vs. *C.stellatus*	< 0.001				0.016	0.049		0.009		
*C.langkawiensis* vs. *C.lekaguli*		0.014		< 0.001		0.042		< 0.001		
*C.langkawiensis* vs. *C.stellatus*	0.001			0.003	< 0.001	0.001		0.002	< 0.001	
*C.lekaguli* vs. *C.stellatus*	< 0.001	< 0.001	0.002		< 0.001	0.042		0.030		

### ﻿Taxonomic hypotheses

According to the concordant results of the phylogenetic analyses, PCA, ANOVA, PERMANOVA, PCAtest and diagnostic morphological characters (see “Comparison”), the populations from Thung Wa and La-ngu Districts, Satun Province are distinctly separated from other species of the *C.pulchellus* group and each other. We, therefore, hypothesise that they represented distinct unnamed species and are described below.

### ﻿Taxonomy

#### ﻿ Cyrtodactylus sungaiupe sp. nov.

Taxon classificationAnimaliaSquamataGekkonidae

016FBB3D-0A29-5AEB-A616-145EF7A305E8

https://zoobank.org/ABB055B2-3790-4DF5-8811-E7CC317AC937

**Figure 5.**
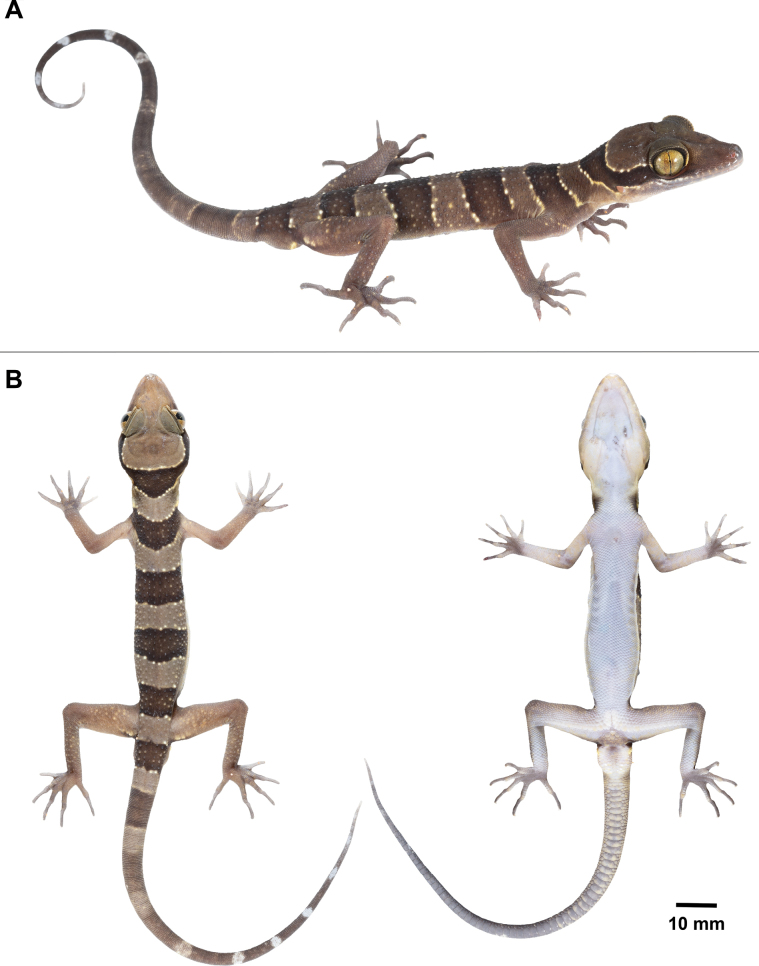
Adult male holotype of *Cyrtodactylussungaiupe* sp. nov. (ZMKU R 01009) from the type locality in Thung Wa Subdistrict, Thung Wa District, Satun Province, Thailand **A** dorsolateral view of specimen in life **B** dorsal and ventral views immediately after euthanasia.

**Figure 6.**
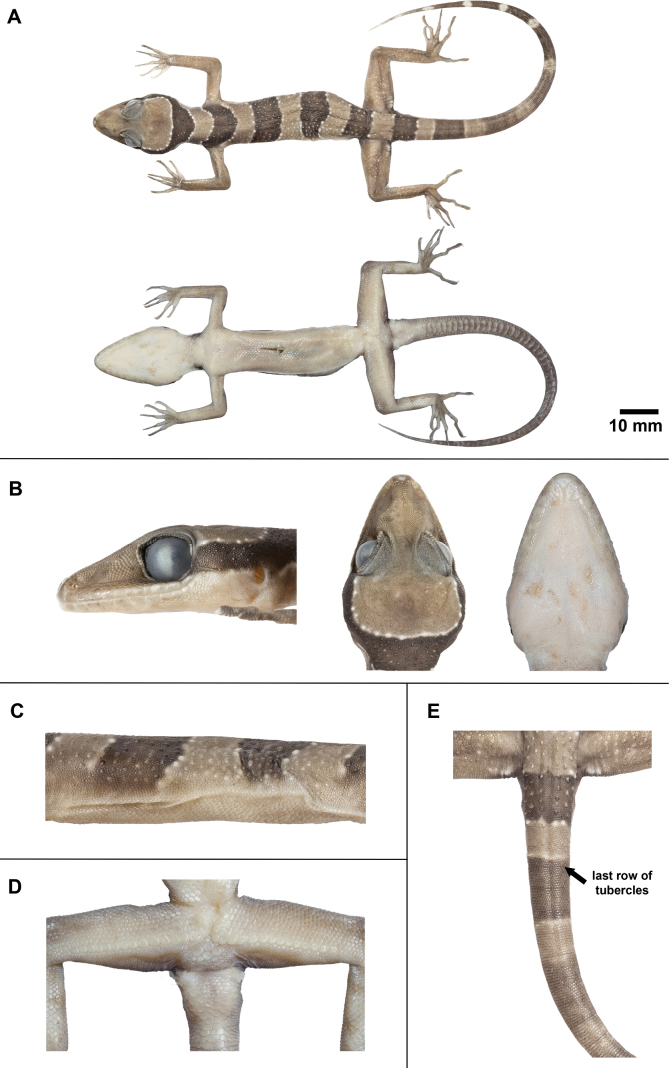
Adult male holotype of *Cyrtodactylussungaiupe* sp. nov. (ZMKU R 01009) in preservation **A** dorsal and ventral views of body **B** head dimensions showing lateral, dorsal and ventral views **C** ventrolateral fold on the left side **D** precloacal depression and pore-bearing femoroprecloacal scales **E** dorsal view of tail showing last row of tubercles.

**Figure 7.**
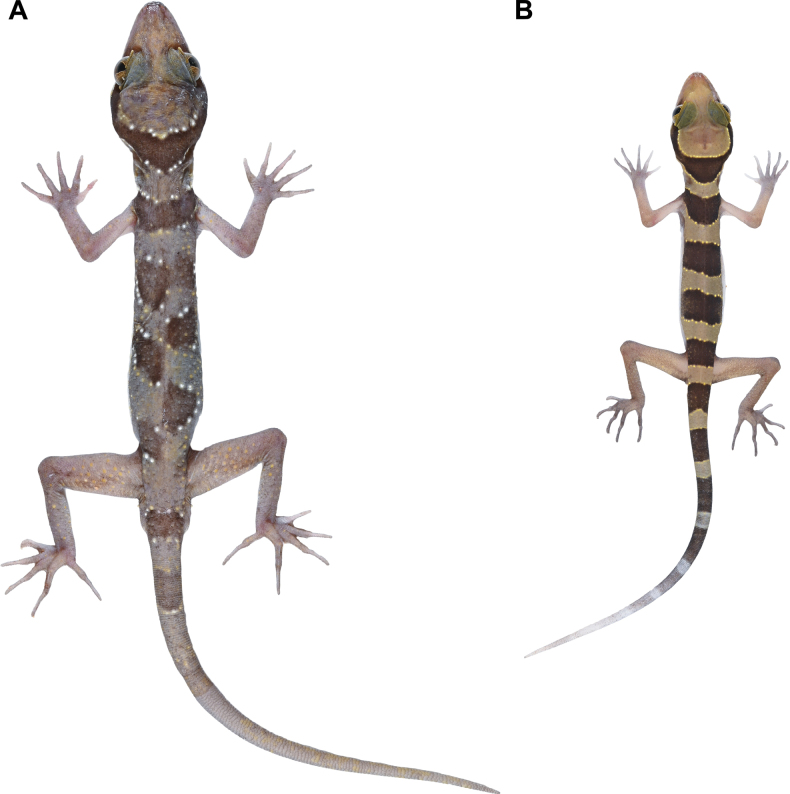
Variation in colouration and banding pattern of *Cyrtodactylussungaiupe* sp. nov. immediately after euthanasia **A** male paratype (ZMKU R 01010) showing disconnected bands on dorsal **B** juvenile (ZMKU R 01013) having light-yellow on the body and bearing white tail tip.

Figs 5 , 6 , 7 Thung Wa Bent-toed Gecko

##### Type material.

***Holotype*.** Adult male (ZMKU R 01009, Figs 5, 6) collected from Thailand, Satun Province, Thung Wa District, Thung Wa Subdistrict (7°05.865'N, 99°58.506'E; 58 m a.s.l.), on 29 April 2022 by Korkhwan Termprayoon, Akrachai Aksornneam, Attapol Rujirawan, and Siriporn Yodthong.

***Paratypes*.** Two adult males (ZMKU R 01004–01005) and two adult females (ZMKU R 01007–01008), same data as holotype, except collected on 12 May 2019, by Korkhwan Termprayoon, Attapol Rujirawan, Natee Ampai, Piyawan Puanprapai and Siriporn Yodthong. One adult male (ZMKU R 01010) and two adult females (ZMKU R 01011–01012), same data as holotype.

##### Referred specimens.

ZMKU R 01006 (immature male), same data as holotype, except collected on 12 May 2019, by Korkhwan Termprayoon, Attapol Rujirawan, Natee Ampai, Piyawan Puanprapai and Siriporn Yodthong. ZMKU R 01013–01014 (two juveniles), same data as holotype.

##### Diagnosis.

*Cyrtodactylussungaiupe* sp. nov. can be distinguished from all other species of the *C.pulchellus* group by a combination of the following characters: (1) SVL 89.7–102.7 mm in adult males (*n* = 4), 87.3–104.6 mm in adult females (*n* = 4); (2) 12–16 supralabial and 10–13 infralabial scales; (3) weak tuberculation on body; (4) no tubercles on ventral surfaces of forelimbs, gular region or in ventrolateral body folds; (5) 30–38 paravertebral tubercles; (6) 19–22 longitudinal rows of dorsal tubercles; (7) 32–40 rows of ventral scales; (8) 20–24 subdigital lamellae on the fourth toe; (9) 29–34 femoroprecloacal pores in adult males; (10) absence of precloacal pores in adult females; (11) deep precloacal groove in males; (12) absence of scattered pattern of white tubercles on dorsum; (13) four dark dorsal body bands; (14) nine or twelve dark caudal bands on original tail; (15) light caudal bands in adults infused with dark pigmentation; (16) caudal tubercles extended 1/8–1/10 of anterior portion of tail and (17) posterior portion of tail in hatchlings and juveniles white.

##### Description of holotype.

Adult male SVL; 89.7 mm; head moderate in length (HL/SVL 0.29) and wide (HW/HL 0.65), flattened (HD/HL 0.37), distinct from neck and triangular in dorsal profile; lores concave anteriorly, inflated posteriorly; frontal and prefrontal regions concave; canthus rostralis rounded anteriorly; snout elongated (ES/HL 0.40), rounded in dorsal profile, laterally constricted; eye large (ED/HL 0.22); ear opening elliptical, moderate in size (EL/HL 0.07), obliquely orientated; eye to ear distance slightly greater than diameter of eye; rostral rectangular, divided dorsally by an inverted Y-shaped furrow, bordered posteriorly by left and right supranasals and internasal, bordered laterally by first supralabials; external nares bordered anteriorly by rostral, dorsally by a large anterior supranasal, posteriorly by two postnasals, ventrally by first supralabial; 9/10 (left/right) rectangular supralabials extending to below mid-point of eye, 13/15 to below the posterior margin of the eye-ball, decreasing abruptly just posterior to mid-point of eye; 8/7 infralabials extending to below mid-point of eye, 13/11 to upturn the labial margin, decreasing gradually in size posteriorly; scales of rostrum and lores slightly raised, larger than granular scales on top of head and occiput, those on posterior portion of canthus rostralis slightly larger; scales on top of head and occiput intermixed with rounded, small tubercles; dorsal superciliaries elongate, smooth, largest anteriorly; mental triangular, 2.5 mm in width, 3.3 mm in length, bordered laterally by first infralabials and posteriorly by left and right, trapezoidal postmentals which contact medially for approximately 50% of their length; one row of slightly enlarged, elongate sublabials extending posteriorly to the sixth (left) and seventh (right) infralabials; small, granular, gular scales grading posteriorly into larger, flat, smooth, imbricate, pectoral and ventral scales.

Body relatively short (AG/SVL 0.47) with well-defined, non-tuberculate, ventrolateral folds; dorsal scales small, granular, interspersed with low, regularly arranged, weakly-keeled tubercles, smaller intervening tubercles occasionally present; tubercles extend from occiput to base of tail, but not further than 1/10 of tail; tubercles on occiput and nape relatively small, those on body largest; approximately 19 longitudinal rows of tubercles at mid-body; 36 paravertebral tubercles; 37 flat imbricate ventral scales between ventrolateral body folds; ventral scales larger than dorsal scales; precloacal scales large, smooth; deep precloacal groove.

Forelimbs moderately slender, relatively short (FL/SVL 0.17); dorsal scales on forelimbs raised, granular, larger than those on body; dorsal scales on forearm intermixed with enlarged, subconical and weakly-keeled tubercles, brachium without tubercles; scales of ventral surface of forearm flat, subimbricate, tubercles absent; palmar scales small, weakly rounded; digits well-developed, inflected at basal, interphalangeal joints; 19/19 (left/right) subdigital lamellae on the fourth finger, 6/6 proximal subdigital lamellae rectangular, broadly expanded proximal to joint inflection, 13/13 distal subdigital lamellae slightly expanded distal to inflection becoming gradually more expanded near the claw; claws well-developed, sheathed by a dorsal and ventral scale; hind limbs more robust than forelimbs, moderate in length (TBL/SVL 0.20), enlarged, subconical, weakly-keeled tubercles on dorsal surface of legs separated by smaller juxtaposed scales; ventral scales of thigh flat, smooth, imbricate, larger than dorsal granular scales; ventral, tibial scales flat, smooth, imbricate; a single row of 35 enlarged femoroprecloacal scales extending nearly from knee to knee through precloacal region where they are continuous with enlarged, pore-bearing precloacal scales; 33 contiguous pore-bearing femoroprecloacal scales, forming an inverted T bearing a deep, precloacal groove (Fig. 6D); six pore-bearing scales bordering groove (three on each side); postfemoral scales immediately posterior to enlarged scale row small, nearly granular, forming an abrupt union with postfemoral scales on posteroventral margin of thigh; plantar scales weakly rounded to flat; 20/20 (left/right) subdigital lamellae on fourth toe, 7/7 proximal subdigital lamellae rectangular, broadly expanded proximal to joint inflection, 13/13 distal subdigital lamellae slightly expanded distal to inflection becoming gradually more expanded near the claw; claws well-developed, sheathed by a dorsal and ventral scale.

Original tail 122.5 mm in length, slightly longer than SVL (TL/SVL = 1.37), 6.4 mm in width at base, tapering to a point; dorsal scales of tail flat, squarish; original portion segmented, approximately 7–8 transverse scales rows per segment; one transverse row of four dorsal tubercles on posterior margin of 1^st^ segment and one tubercle on 2^nd^ segment; caudal tubercles extended 1/10 of anterior portion of original tail (Fig. 6E); subcaudal region bearing large median row of transverse scales; shallow dorsal and lateral caudal furrow; base of tail bearing hemipenial swellings; two rows of (1+4)L/(2+3)R medium-sized postcloacal tubercles on each hemipenial swelling; postcloacal scales smooth, flat, large, imbricate.

##### Colouration in life

**(Fig. 5).** Ground colour of head, body, and limbs light-brown; superciliaries yellow anteriorly and posteriorly; supralabial and infralabial scales light-brown with off-white markings posteriorly; wide, dark-brown nuchal band edged anteriorly and posteriorly by thin, yellowish lines bearing tubercles extending from posterior margin of one eye to posterior margin of another eye; four similar dark-brown body bands between nuchal loop and hind limb insertions edged anteriorly and posteriorly by broken, thin, creamy-white to yellow lines bearing tubercles, first band terminating at shoulders, second and third bands terminating just dorsal to ventrolateral folds, the fourth band terminating at femurs; dark body bands slightly larger than light-coloured interspaces; one additional dark-brown band posterior to hind limbs (postsacral band); ventral surfaces of head, abdomen and limbs greyish-white; creamy pale yellow postcloacal tubercles; tail bearing nine dark bands separated by ten light-brown (anteriorly) to white (posteriorly) bands, white caudal band infused with dark pigmentation; subcaudal region off-white anteriorly, becoming darker posteriorly.

##### Colouration in preservative

**(Fig. 6).** The overall colour pattern of head, body, limbs and tail similar to that in life with some fading. Ground colour of head, body, limbs and dorsum tan; dark bands on dorsum and tail brown; yellow-coloured tuberculation on dorsum fading to off-white or white; light-beige coloured on the ventral surface.

##### Variation.

Meristic and morphometric data for the type series and referred specimens of *Cyrtodactylussungaiupe* sp. nov. are given in Tables 6, 7 and Suppl. material 3. All paratypes and referred specimens resemble the holotype in general aspects of morphology with variations in colouration and banding pattern. All specimens have continuous dark body bands, except one specimen (ZMKU R 01010) where the second to fourth dorsal bands do not connect on the mid-line and are offset (Fig. 7A). Dorsal scale on brachium of two specimens (ZMKU R 01010 and ZMKU R 01012) intermixed with tubercles, whereas absent in the holotype and other specimens. ZMKU R 01005 has dark marking on left femur. Three male paratypes have discontinuous pore-bearing femoroprecloacal scales; ZMKU R 01004 has three poreless scales (one on the left and two on the right), ZMKU R 01005 has one enlarged and one small poreless scales on the left and ZMKU R 01010 has two poreless scales (one on each side). Last row of caudal tubercles of paratypes (ZMKU R 01006 and ZMKU R 01008) extending to 4^th^–5^th^ segment of original portion (to 2^nd^ light caudal band), approximately 1/8 of anterior portion of original tail. Juveniles (ZMKU R 01013–01014) have body pattern similar to adults, but less prominent tuberculation, light-yellow ground colour of body, edged anteriorly and posteriorly by yellowish lines bearing tubercles, the original tail has approximately six or ten dark caudal bands, the posterior portion of tail is white (Fig. 7B).

**Table 6.** Descriptive measurements (millimetres), meristic (left/right) and non-meristic characters of the type series of *Cyrtodactylussungaiupe* sp. nov. Key: H = holotype, P = paratype, M = male, F = female; Or = original tail, Br = broken, Re = regenerated; / = data unavailable or inapplicable; L = left, R = right. Abbreviations are defined in Materials and methods.

	ZMKU R 01009	ZMKU R 01004	ZMKU R 01005	ZMKU R 01010	ZMKU R 01007	ZMKU R 01008	ZMKU R 01011	ZMKU R 01012
Type series	H	P	P	P	P	P	P	P
Sex	M	M	M	M	F	F	F	F
SVL	89.7	102.7	97.5	98.2	104.1	100.6	87.3	104.6
Tail condition	Or	Re	Re	Re	Br	Or	Re	Re
TL	122.5	98.6	134.5	103.1	112.8	125.1	69.1	130.5
TW	6.4	7.6	7.1	6.0	6.9	6.7	4.9	6.4
FL	15.4	16.4	16.1	15.3	17.0	15.8	14.3	16.4
TBL	17.6	20.1	18.0	18.9	20.6	18.6	16.8	20.1
AG	41.9	53.9	48.6	45.7	51.9	51.3	41.6	52.9
HL	26.3	29.4	27.6	28.7	30.4	27.8	24.9	30.4
HW	17.0	19.2	18.4	18.7	19.5	17.6	15.8	19.5
HD	9.7	11.7	10.9	10.8	11.1	10.9	9.4	10.9
ED	5.9	6.4	6.8	7.0	7.0	7.0	5.9	7.1
EE	6.8	8.3	8.1	7.3	7.7	7.4	6.8	7.9
ES	10.5	11.5	11.3	11.3	12.2	11.4	10.1	11.6
EN	8.0	9.0	8.5	8.8	9.6	8.8	7.8	9.0
IO	6.0	6.3	7.0	6.2	6.7	6.1	5.6	6.5
EL	1.9	2.4	2.3	2.5	2.6	2.5	2.2	2.4
IN	3.4	3.2	3.2	3.6	3.6	3.3	3.0	3.5
SL	13/15	13/12	14/13	15/15	14/15	12/12	16/14	14/15
SL-mid-eye	9/10	10/9	9/8	11/10	9/11	9/10	11/10	9/10
IL	13/11	11/11	10/11	12/13	13/13	12/11	11/11	11/13
IL-mid-eye	8/7	8/8	7/8	8/9	8/8	7/8	8/8	7/8
PVT	36	34	36	33	33	38	31	30
LRT	19	21	20	20	20	22	19	19
VS	37	37	37	34	40	37	33	35
4FLU	13/13	13/15	12/11	12/12	14/14	13/13	14/14	15/15
4FLE	6/6	7/6	6/5	5/5	6/6	6/6	6/6	6/6
4FL	19/19	20/21	18/17	17/17	20/20	19/19	20/20	21/21
4TLU	13/13	15/15	13/13	13/13	14/14	15/15	15/15	16/16
4TLE	7/7	8/7	7/7	7/7	7/7	8/7	8/7	8/8
4TL	20/20	23/22	20/20	20/20	21/21	23/22	23/22	24/24
FPP in males	33	34	29	31	/	/	/	/
No of pore-bearing scales on precloacal groove	6 (3L/3R)	5 (2L/3R)	6 (3L/3R)	6 (3L/3R)	/	/	/	/
PCT rows	2L/2R	2L/2R	1L/2R	2L/2R	/	/	/	/
No of PCT per row	(1+4)L/(2+3)R	(2+4)L/(3+4)R	(3)L/(2+3)R	(2+3)L/(2+4)R	/	/	/	/
BB	4	4	4	4	4	4	4	4
LCB	10	/	/	/	/	8	/	/
DCB	9	/	/	/	/	9	/	/
Body band/ interspace ratio	1.50	1.30	0.66	/	1.43	1.40	1.01	1.17
Deep precloacal groove in male	Yes	Yes	Yes	Yes	/	/	/	/
Femoroprecloacal pores continuous	Yes	No	No	No	/	/	/	/
Tuberculation	Weak	Weak	Weak	Weak	Weak	Weak	Weak	Weak
Tubercles on ventral surface of forelimb	No	No	No	No	No	No	No	No
Tubercles in gular region	No	No	No	No	No	No	No	No
Ventrolateral fold tuberculate	No	No	No	No	No	No	No	No
Dorsum bearing scattered pattern of white tubercles	No	No	No	No	No	No	No	No
Adult posterior caudal region white	No	/	/	/	/	No	/	/
White caudal bands in adults immaculate	No	/	/	/	/	No	/	/
Portion of caudal tubercles on original tail	1/10	/	/	/	/	1/8	/	/

**Table 7.** Descriptive meristic (left/right) and non-meristic characters of referred specimens of *Cyrtodactylussungaiupe* sp. nov. Key: RF = referred specimens, IM-M = immature male, J = juvenile; / = data unavailable or inapplicable. Abbreviations are defined in Materials and methods.

	ZMKU R 01006	ZMKU R 01013	ZMKU R 01014
	RF	RF	RF
Age	IM-M	J	J
SVL	81.2	59.8	67.3
SL	13/13	15/14	15/14
SL-mid-eye	9/9	10/11	11/10
IL	11/10	10/11	11/12
IL-mid-eye	7/7	6/8	8/9
PVT	34	34	33
LRT	20	19	20
VS	38	36	32
4FLU	13/12	15/15	13/13
4FLE	6/7	6/6	6/5
4FL	19/19	21/21	19/18
4TLU	13/13	16/16	14/14
4TLE	8/8	7/7	8/8
4TL	21/21	23/23	22/22
BB	4	4	4
LCB	13	/	/
DCB	12	/	/
Body band/ interspace ratio	1.11	1.64	1.17
Tuberculation	Weak	Weak	Weak
Tubercles on ventral surface of forelimb	No	No	No
Tubercles in gular region	No	No	No
Ventrolateral fold tuberculate	No	No	No
Dorsum bearing scattered pattern of white tubercles	No	No	No
Hatchlings/ juveniles with white tail tip	/	Yes	Yes
Portion of caudal tubercles on original tail	1/8	/	/

##### Distribution.

**Figure 8.**
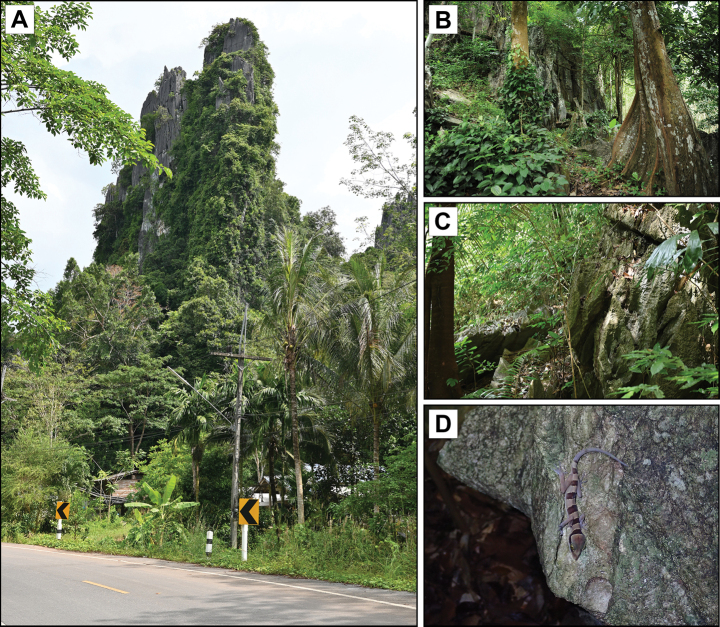
Habitat of *Cyrtodactylussungaiupe* sp. nov. at the type locality in Thung Wa Subdistrict, Thung Wa District, Satun Province, Thailand **A** landscape view of karst tower **B** vegetation structure and **C** karst boulders surrounding karst body **D** immature female (not collected) on karst boulder.

*Cyrtodactylussungaiupe* sp. nov. is currently known from an unnamed karst formation in Thung Wa Subdistrict, Thung Wa District, Satun Province, Thailand (Figs 1, 8).

##### Natural history.

All individuals of *Cyrtodactylussungaiupe* sp. nov. were collected from karst forest (Fig. 8) at the type locality which was surrounded by rubber plantations. Specimens were collected on October 2016 between 1900 and 2000 h with temperature 26.3 °C and relative humidity 90.3%, and on April 2022 between 1900 and 2030 h with temperature 28.3 °C and relative humidity 86.0%. The specimens were found on small rocks, karst boulders, adjacent vegetation and on the forest floor. The holotype (ZMKU R 01009) was collected from the branch of a shrub approximately 1.3 m above the ground. Four specimens (ZMKU R 01004, ZMKU R 01006, ZMKU R 01010, ZMKU R 01013) were found on karst boulders. Three female specimens (ZMKU R 01008, ZMKU R 01011–01012) were collected from tree trunks. One adult male (ZMKU R 01005) was collected off a fallen dry stick and one adult female (ZMKU R 01007) was found on the forest floor covered with leaf litter. A juvenile (ZMKU R 01014) was taken from a perch on a small rock near the ground.

A gravid female (ZMKU R 01008) carrying two eggs (externally visible) was found on a tree trunk in October 2016. Two juveniles (ZMKU R 01013–01014) were collected during April 2022. Other lizard species found in this area, include *Gehyramutilata* (Wiegmann, 1834) and *Gekkogecko* (Linnaeus, 1758).

##### Etymology.

The specific epithet *sungaiupe* is derived from the old name of Thung Wa District (Sungai Upe District), the type locality of the new species.

##### Comparison.

*Cyrtodactylussungaiupe* sp. nov. can be distinguished from other species in the *C.pulchellus* group by having a combination of weak tuberculation on the body; no tubercles on ventral surface of forelimbs, gular region or in ventrolateral body folds; 12–16 supralabial scales; 30–38 paravertebral tubercles; 19–22 longitudinal tubercle rows; 32–40 ventral scales; 29–34 femorprecloacal pores in males; deep precloacal groove in males; nine or twelve dark caudal bands on original tail; light caudal bands on original tail, infused with dark pigmentation in adults; caudal tubercles extended 1/8–1/10 of anterior portion of tail; and juveniles with white tail tip. Additional comparisons between *Cyrtodactylussungaiupe* sp. nov. and other species in the *C.pulchellus* group are in Suppl. material 4.

*Cyrtodactylussungaiupe* sp. nov. is a member of Clade A which comprises *C.astrum*, *C.dayangbuntingensis*, *C.langkawiensis*, *C.lekaguli* and *C.stellatus*. *Cyrtodactylussungaiupe* sp. nov. differs from those five species by uncorrected pairwise distances of ND2 of 8.02–10.69% (Table 2). It differs from *C.astrum* by having smaller maximum SVL of 104.6 mm (vs. 108.3 mm); absence of scattered pattern of white tubercles on dorsum (vs. present); nine or twelve dark caudal bands on the original tail (vs. 13 or 14); caudal tubercles extending between 1/8 and 1/10 of anterior portion of tail (vs. ≥ 1/3 of the tail); and having statistically significant different mean values of mensural characters of FL_adj_, TBL_adj_, AG_adj_, HW_adj_, HD_adj_, EE_adj_, ES_adj_, EN_adj_ and IN_adj_ (*p* < 0.001–0.043; Table 5). It can be further separated from *C.astrum* in having statistically significant different mean values of meristic characters of SL, IL, PVT and LRT (*p* < 0.001–0.011; Table 5).

*Cyrtodactylussungaiupe* sp. nov. differs from *C.dayangbuntingensis* by having larger maximum SVL of 104.6 mm (vs. 99.0 mm); absence of scattered pattern of white tubercles on dorsum (vs. present); caudal tubercles extending between 1/8 and 1/10 of anterior portion of tail (vs. 1/5 of the tail); and one to two rows of postcloacal tubercles (vs. up to three rows). Additionally, PCA showed that *Cyrtodactylussungaiupe* sp. nov. is clearly separated in morphospace from *C.dayangbuntingensis* (Fig. 3).

*Cyrtodactylussungaiupe* sp. nov. morphologically differ from *C.langkawiensis* by having larger maximum SVL of 104.6 mm (vs. 99.8 mm); and caudal tubercles extending between 1/8 and 1/10 of anterior portion of tail (vs. ≥ 1/3 of the tail); and having statistically significant different mean values of mensural characters of AG_adj_, HL_adj_, HW_adj_ and IN_adj_ (*p* < 0.001–0.033; Table 5). It can be further separated from *C.langkawiensis* in having statistically significant different mean values of meristic characters of SL, IL, PVT, LRT and VS (*p* < 0.001–0.046; Table 5).

*Cyrtodactylussungaiupe* sp. nov. differ from *C.lekaguli* by having smaller maximum SVL of 104.6 mm (vs. 108.5 mm); and caudal tubercles extending between 1/8 and 1/10 of anterior portion of tail (vs. ≥ 1/3 of the tail); and having statistically significant different mean values of mensural characters of HW_adj_, HD_adj_, EE_adj_, IO_adj_ and EL_adj_ (*p* < 0.001–0.008; Table 5). It can be further separated from *C.lekaguli* in having statistically significant different mean values of meristic characters of SL, IL and LRT (*p* < 0.001–0.006; Table 5).

*Cyrtodactylussungaiupe* sp. nov. differ from *C.stellatus* by having larger maximum SVL of 104.6 mm (vs. 96.1 mm); absence of scattered pattern of white tubercles on dorsum (vs. present); absence of precloacal in female (vs. present); and having statistically significant different mean values of mensural characters of SVL, FL_adj_, TBL_adj_, AG_adj_, HL_adj_, HW_adj_, HD_adj_, EE_adj_, ES_adj_ and EN_adj_ (*p* < 0.001–0.035; Table 5). It can be further separated from *C.stellatus* in having statistically significant different mean values of meristic characters of PVT (*p* = 0.003; Table 5).

#### ﻿ Cyrtodactylus wangkhramensis sp. nov.

Taxon classificationAnimaliaSquamataGekkonidae

F7410CEC-A0A3-54B4-84B2-06C704D5D478

https://zoobank.org/D2615DAF-F982-41C0-85A1-4C8D93816E99

**Figure 9.**
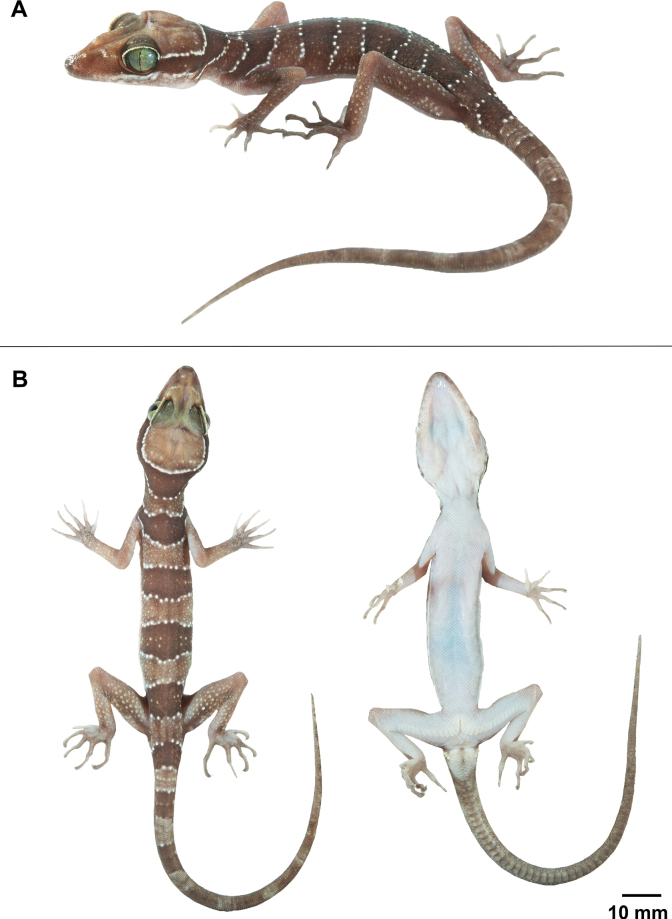
Adult male holotype of *Cyrtodactyluswangkhramensis* sp. nov. (ZMKU R 01018) from the type locality at Tham Wangkhram, La-ngu District, Satun Province, Thailand **A** dorsolateral view of specimen in life **B** dorsal and ventral views immediately after euthanasia.

**Figure 10.**
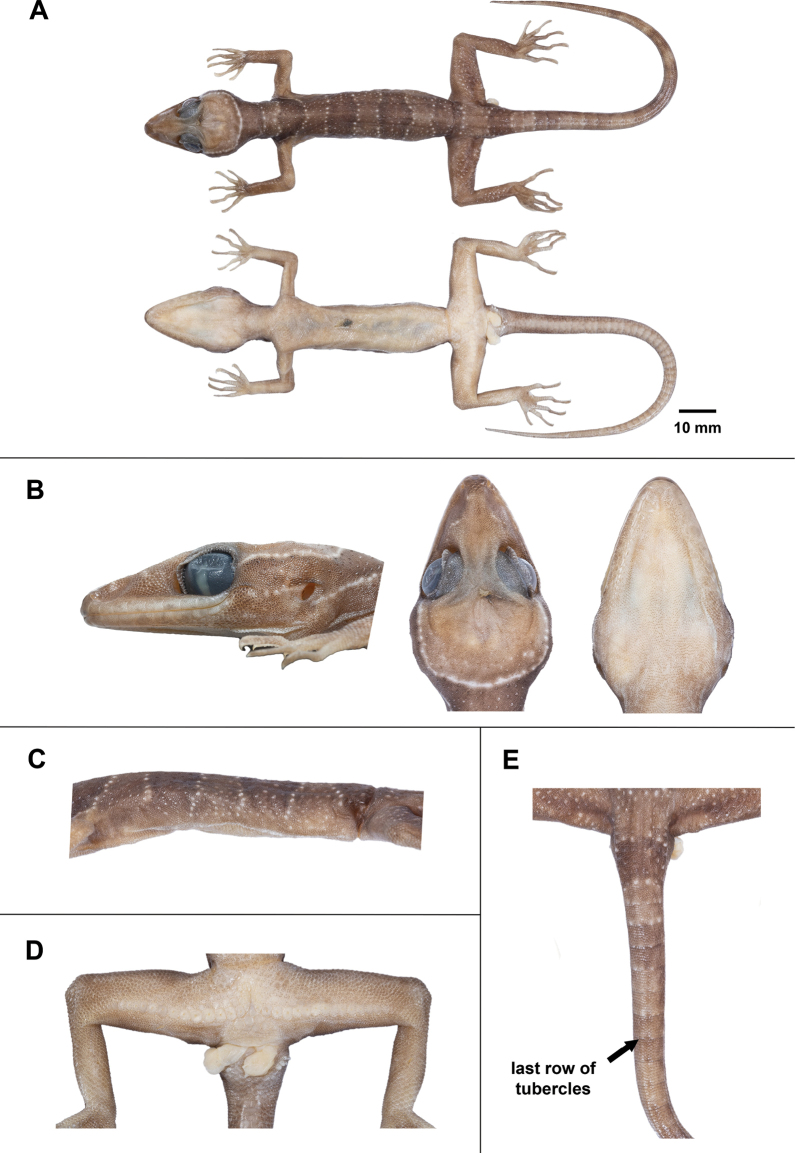
Adult male holotype of *Cyrtodactyluswangkhramensis* sp. nov. (ZMKU R 01018) in preservation **A** dorsal and ventral views of body **B** head dimensions showing lateral, dorsal and ventral views **C** ventrolateral fold on the left side **D** precloacal depression and pore-bearing femoroprecloacal scales **E** dorsal view of tail showing last row tubercles.

**Figure 11.**
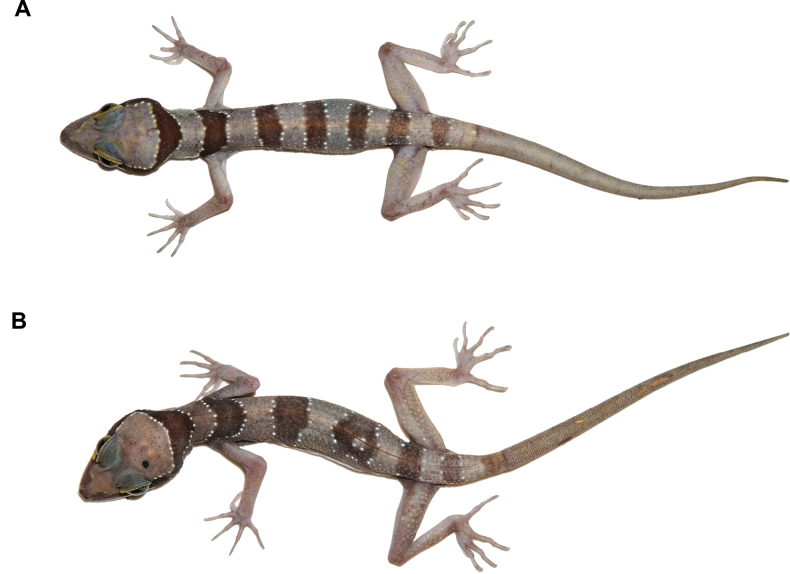
Variation in banding pattern of *Cyrtodactyluswangkhramensis* sp. nov. immediately after euthanasia **A** adult male (ZMKU R 01016) having five dark body bands on dorsum **B** adult male (ZMKU R 01026) showing irregular pattern on the 3^rd^ body band.

Figs 9 , 10 , 11 Wangkhram Bent-toed Gecko

##### Type material.

***Holotype*.** Adult male (ZMKU R 01018, Figs 9, 10) collected from Thailand, Satun Province, La-ngu District, Khao Khao Subdistrict, Tham (= cave) Wangkhram (6°56.324'N, 99°48.920'E; 0 m a.s.l.), on 13 March 2019 by Korkhwan Termprayoon, Anchalee Aowphol, Attapol Rujirawan, Natee Ampai and Siriporn Yodthong.

***Paratypes*.** Eleven paratypes, four adult males (ZMKU R 01015–01017 and ZMKU R 01019) and seven adult females (ZMKU R 01020–01026), same data as holotype.

##### Diagnosis.

*Cyrtodactyluswangkhramensis* sp. nov. can be distinguished from all other species of the *C.pulchellus* group by a combination of the following characters: (1) SVL 87.4–95.5 mm in adult males (*n* = 5), 89.4–98.8 mm in adult females (*n* = 7); (2) 11–14 supralabial and 9–13 infralabial scales; (3) weak tuberculation on body; (4) no tubercles on ventral surfaces of forelimbs, gular region or in ventrolateral body folds; (5) 28–35 paravertebral tubercles; (6) 19–21 longitudinal rows of dorsal tubercles; (7) 34–40 rows of ventral scales; (8) 18–22 subdigital lamellae on the fourth toe; (9) 32–36 femoroprecloacal pores in adult males; (10) absence of precloacal pores in adult females; (11) deep precloacal groove in males; (12) absence of scattered pattern of white tubercles on dorsum; (13) four or five dark dorsal body bands; (14) 11 dark caudal bands on original tail; (15) light caudal bands in adults infused with dark pigmentation; and (16) caudal tubercles extending 1/5–1/7 of anterior portion of tail.

##### Description of holotype.

Adult male SVL; 94.2 mm; head moderate in length (HL/SVL 0.28) and wide (HW/HL 0.63), flattened (HD/HL 0.38), distinct from neck and triangular in dorsal profile; lores concave anteriorly, inflated posteriorly; frontal and prefrontal regions deeply concave; canthus rostralis rounded anteriorly; snout elongate (ES/HL 0.41), rounded in dorsal profile, laterally constricted; eye large (ED/HL 0.23); ear opening elliptical, moderate in size (EL/HL 0.08), obliquely orientated; eye to ear distance slightly greater than diameter of eye; rostral rectangular, divided dorsally by an inverted U-shaped furrow, bordered posteriorly by left and right supranasals and large hexagonal internasal, bordered laterally by first supralabials; external nares bordered anteriorly by rostral, dorsally by a large anterior supranasal, posteriorly by two postnasals, ventrally by first supralabial; 8/8 (left/right) rectangular supralabials extending to below mid-point of eye, 12/12 to below the posterior margin of the eye-ball, decreasing abruptly just posterior to mid-point of eye; 7/7 infralabials extending to below mid-point of eye, 11/11 to upturn the labial margin, decreasing gradually in size posteriorly; scales of rostrum and lores slightly raised, larger than granular scales on top of head and occiput, those on posterior portion of canthus rostralis slightly larger; scales on top of head and occiput intermixed with rounded, small tubercles; dorsal superciliaries elongate, smooth, largest anteriorly; mental triangular, 3.60 mm in width, 2.38 mm in length, bordered laterally by first infralabials and posteriorly by left and right, trapezoidal postmentals which contact medially for approximately 45% of their length; one row of slightly enlarged, elongate sublabials extending posteriorly to the seventh (left/right) infralabials; small, granular, gular scales grading posteriorly into larger, flat, smooth, imbricate, pectoral and ventral scales.

Body relatively short (AG/SVL 0.47) with well-defined, non-tuberculate, ventrolateral folds; dorsal scales small, granular, interspersed with low, regularly arranged, weakly-keeled tubercles, smaller intervening tubercles occasionally present; tubercles extending from occiput to base of tail, but not further than 1/5 of tail; tubercles on occiput and nape relatively small, those on body largest; approximately 20 longitudinal rows of tubercles at mid-body; 32 paravertebral tubercles; 35 flat imbricate ventral scales between ventrolateral body folds; ventral scales larger than dorsal scales; precloacal scales large, smooth; deep precloacal groove.

Forelimbs moderately slender, relatively short (FL/SVL 0.17); dorsal scales on forelimbs slightly raised, granular, slightly larger than those on body intermixed with enlarged, subconical and weakly-keeled tubercles; scales of ventral surface of forearm flat, subimbricate, tubercles absent; palmar scales small, weakly rounded; digits well-developed, inflected at basal, interphalangeal joints; 17/17 (left/right) subdigital lamellae on the fourth finger, 6/6 proximal subdigital lamellae rectangular, broadly expanded proximal to joint inflection, 11/11 distal subdigital lamellae slightly expanded distal to inflection becoming gradually more expanded near the claw; claws well-developed, sheathed by a dorsal and ventral scale; hind limbs more robust than forelimbs, moderate in length (TBL/SVL 0.20), enlarged, subconical, weakly-keeled tubercles on dorsal surface of legs separated by smaller juxtaposed scales; ventral scales of thigh flat, smooth, imbricate, larger than dorsal granular scales; ventral, tibial scales flat, smooth, imbricate; a single row of 34 enlarged femoroprecloacal scales extending nearly from knee to knee through precloacal region where they are continuous with enlarged, pore-bearing precloacal scales; 32 contiguous pore-bearing femoroprecloacal scales (Fig. 10D), forming an inverted T bearing a deep, precloacal groove; seven pore-bearing scales bordering groove (four on left and three on right); postfemoral scales immediately posterior to enlarged scale row small, nearly granular, forming an abrupt union with postfemoral scales on posteroventral margin of thigh; plantar scales weakly rounded to flat; 18/18 subdigital lamellae on fourth toe, 7/7 proximal subdigital lamellae rectangular, broadly expanded proximal to joint inflection, 11/11 distal subdigital lamellae slightly expanded distal to inflection becoming gradually more expanded near the claw; claws well-developed, sheathed by dorsal and ventral scales.

Regenerated tail 120.3 mm in length, slightly longer than SVL (TL/SVL = 1.28), 5.4 mm in width at base, tapering to a point; dorsal scales of tail flat, squarish; original portion segmented, 7–9 transverse scales rows per segment; seven transverse rows of 2–6 dorsal tubercles on posterior margin, fewer posteriorly; subcaudal region bearing large median row of transverse scales; regenerated portion of tail covered with small, smooth, rectangular scales dorsally and ventrally; shallow dorsal and lateral caudal furrow extending entire length of original tail; base of tail bearing hemipenial swellings; two rows of (3+2)L/(1+3)R medium-sized postcloacal tubercles on each hemipenial swelling; postcloacal scales smooth, flat, large, imbricate.

##### Colouration in life

**(Fig. 9).** Ground colour of body, limbs, light-brown, those on head lighter; faded, incomplete V-shaped rostral chevron; superciliaries yellow; supralabial and infralabial scales light-brown anteriorly, with off-white-coloured medially to posteriorly; wide, dark-brown nuchal band edged anteriorly and posteriorly by thin, white lines bearing tubercles extending from posterior margin of one eye to posterior margin of another eye; four dark-brown body bands between nuchal loop and hind limb insertions edged anteriorly and posteriorly by broken, thin, white lines formed by a single row of white tubercles, first band terminating at shoulders, second and third bands terminating just dorsal to ventrolateral folds, the fourth band terminating at femurs; dark body bands slightly larger than light-coloured interspaces; creamy pale yellow tubercles on dorsal surfaces of limbs, those on body darker, similar to ground colour; one additional dark-brown band posterior to hind limbs (postsacral band); ventral surfaces of head, abdomen and limbs greyish-white; creamy pale yellow postcloacal tubercles; tail bearing approximately six dark bands separated by six light-brown (anteriorly) to white (posteriorly) bands, white caudal band heavily infused with dark-brown pigmentation on original portion; regenerated portion and subcaudal region tan.

##### Colouration in preservative

**(Fig. 10).** The overall colour pattern of head, body, limbs and tail similar to that in life with some fading. Ground colour of head, body, limbs and dorsum dark tan; dark body bands darker; creamy pale yellow-coloured tuberculation on dorsum fading to off-white; beige coloured on the ventral surface.

##### Variation.

Meristic and morphometric data for the type series of *Cyrtodactyluswangkhramensis* sp. nov. are given in Table 8 and Suppl. material 3. All paratypes resemble the holotype in general aspects of morphology with variations in colouration and banding pattern. Eight specimens have four dark body bands and three specimens (ZMKU R 01016–01017 and ZMKU R 01021) have five dark bands on dorsum (Fig. 11A). Female paratype (ZMKU R 01025) has four dark body bands with an irregular pattern on the third (Fig. 11B). Original tails (ZMKU R 01019–01021) have 11 dark caudal bands and 10–11 light caudal bands. Last row of caudal tubercles of paratypes (ZMKU R 01019–01021) extending to 4^th^–7^th^ segment of original portion, approximately 1/5–1/7 of anterior portion of original tail.

**Table 8.** Descriptive measurements (millimetres), meristic (left/right) and non-meristic characters of the type series of *Cyrtodactyluswangkhramensis* sp. nov. Key: H = holotype, P = paratype, M = male, F = female; Or = original tail, Br = broken, Re = regenerated; / = data unavailable or inapplicable; L = left, R = right. Abbreviations are defined in Materials and methods.

	ZMKU R 01018	ZMKU R 01015	ZMKU R 01016	ZMKU R 01017	ZMKU R 01019	ZMKU R 01020	ZMKU R 01021	ZMKU R 01022	ZMKU R 01023	ZMKU R 01024	ZMKU R 01025	ZMKU R 01026
Type series	H	P	P	P	P	P	P	P	P	P	P	P
Sex	M	M	M	M	M	F	F	F	F	F	F	F
SVL	94.2	89.7	95.5	92.5	87.4	93.4	95.2	98.8	92.3	95.9	93.7	89.4
Tail condition	Re	Re	Re	Br	Or	Or	Or	Re	Re	Br	Re	Re
TL	120.3	122.5	91.8	/	119.5	126.6	120.4	93.9	116.5	51.3	111.2	88.3
TW	5.4	7.4	6.6	/	5.4	5.2	5.8	6.1	5.6	5.8	5.8	5.0
FL	15.6	15.5	15.2	15.7	14.8	15.3	16.2	16.4	15.8	15.5	15.3	15.0
TBL	18.5	18.5	18.7	17.7	18.1	17.4	18.1	18.8	18.6	18.7	18.4	17.2
AG	44.6	45.5	45.1	44.8	41.1	47.1	47.0	49.2	45.1	47.1	45.1	43.1
HL	26.4	26.8	27.5	27.5	25.8	26.8	27.5	28.8	27.8	28.6	27.9	26.4
HW	16.7	17.5	17.5	17.6	16.1	17.2	17.8	18.3	17.8	17.5	17.3	16.5
HD	10.0	11.5	10.6	10.5	9.8	10.1	10.7	10.8	10.1	10.2	10.7	9.9
ED	6.2	6.7	7.0	6.7	6.3	6.1	6.7	6.5	6.7	6.5	6.3	6.1
EE	7.0	7.1	7.1	7.0	6.9	6.7	7.0	7.0	6.8	6.5	6.9	6.7
ES	10.7	11.1	11.3	10.4	10.3	10.2	11.2	11.5	10.9	11.0	10.9	10.2
EN	8.2	8.5	8.6	8.1	8.0	7.8	8.5	8.9	8.3	8.4	8.3	8.0
IO	5.7	6.2	6.2	6.5	5.4	5.9	6.4	6.4	5.6	6.0	6.1	6.2
EL	2.0	2.1	1.6	1.9	2.1	1.9	2.3	2.8	1.9	2.0	2.1	2.2
IN	3.0	3.3	2.7	2.8	2.7	3.1	3.4	3.5	3.4	3.3	3.1	3.0
SL	12/12	13/12	13/13	13/13	12/12	11/13	13/13	12/13	12/12	13/12	12/14	13/13
SL-mid-eye	8/8	7/7	10/9	9/9	9/9	7/9	9/9	9/9	9/9	9/9	9/10	10/10
IL	11/11	11/11	11/13	11/11	11/11	11/11	12/10	11/11	11/11	11/11	11/11	9/11
IL-mid-eye	7/7	9/9	7/8	8/8	7/8	7/7	9/7	9/7	7/8	8/8	7/8	7/8
PVT	32	35	35	32	33	34	32	31	32	35	28	34
LRT	20	21	21	21	20	21	20	21	19	21	20	19
VS	35	40	34	35	37	35	37	36	34	37	36	35
4FLU	11/11	11/12	13/13	11/11	11/12	12/12	13/12	12/12	13/13	13/13	12/12	12/12
4FLE	6/6	6/6	6/6	6/6	6/6	6/6	6/6	6/6	6/6	6/6	6/6	6/6
4FL	17/17	17/18	19/19	17/17	17/18	18/18	19/18	18/18	19/19	19/19	18/18	18/18
4TLU	11/11	12/12	14/14	13/12	12/12	13/13	13/13	13/13	13/13	14/13	12/12	13/13
4TLE	7/7	7/8	7/7	8/8	7/7	8/8	8/8	8/8	8/8	8/7	7/7	8/8
4TL	18/18	19/20	21/21	21/20	19/19	21/21	21/21	21/21	21/21	22/20	19/19	21/21
FPP in males	32	33	33	36	32	/	/	/	/	/	/	/
No of pore-bearing scales on precloacal groove	7 (4L/3R)	6 (3L/3R)	6 (3L/3R)	6 (3L/3R)	7 (3L/4R)	/	/	/	/	/	/	/
PCT rows	2L/2R	2L/2R	2L/1R	2L/2R	2L/2R	/	/	/	/	/	/	/
No of PCT per row	(3+2)L/(1+3)R	(2+3)L/(1+4)R	(1+4)L/(3)R	(3+4)L/(3+4)R	(2+4)L/(3+4)R	/	/	/	/	/	/	/
BB	4	4	5	5	4	4	5	4	4	4	4	4
LCB	/	/	/	/	10	11	10	/	/	/	/	/
DCB	/	/	/	/	11	11	11	/	/	/	/	/
Body band/ interspace ratio	1.70	1.09	0.89	1.31	1.18	1.05	/	0.93	0.82	1.25	1.01	1.38
Deep precloacal groove in male	Yes	Yes	Yes	Yes	Yes	/	/	/	/	/	/	/
Femoroprecloacal pores continuous	Yes	Yes	Yes	Yes	Yes	/	/	/	/	/	/	/
Tuberculation	Weak	Weak	Weak	Weak	Weak	Weak	Weak	Weak	Weak	Weak	Weak	Weak
Tubercles on ventral surface of forelimb	No	No	No	No	No	No	No	No	No	No	No	No
Tubercles in gular region	No	No	No	No	No	No	No	No	No	No	No	No
Ventrolateral fold tuberculate	No	No	No	No	No	No	No	No	No	No	No	No
Dorsum bearing scattered pattern of white tubercles	No	No	No	No	No	No	No	No	No	No	No	No
Adult posterior caudal region white	/	/	/	/	No	No	No	/	/	/	/	/
White caudal bands in adults immaculate	/	/	/	/	No	No	No	/	/	/	/	/
Portion of caudal tubercles on original tail	/	/	/	/	1/5	1/7	1/7	/	/	/	/	/

##### Distribution.

**Figure 12.**
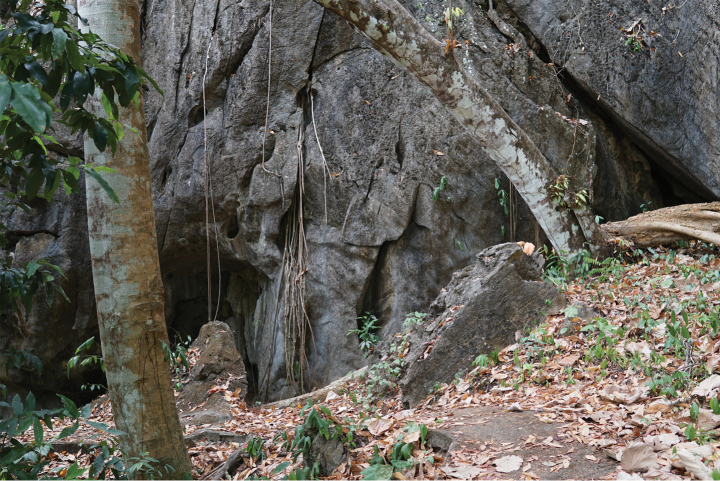
Habitat of *Cyrtodactyluswangkhramensis* sp. nov. at the type locality, Tham Wangkhram, La-ngu District, Satun Province, Thailand.

*Cyrtodactyluswangkhramensis* sp. nov. is currently known from the type locality at Tham Wangkhram, Khao Khao Subdistrict, La-ngu District, Satun Province, Thailand (Figs 1, 12).

##### Natural history.

Type series of *Cyrtodactyluswangkhramensis* sp. nov. was collected from a karst formation at night (1900–2100 h) during March 2019. Eleven specimens were captured from substrates surrounding the karstic area (karst wall, crevices, karst boulders, plantation and concrete poles). Ambient temperature was 25.7 °C and relative humidity was 90.7%. The male holotype (ZMKU R 01018) was found on a termite mound adjacent to a karst outcrop. Five individuals (ZMKU R 01016–01017, ZMKU R 01020–01021 and ZMKU R 01023) were found on a karst wall. One specimen ZMKU R 01015 was found perched on a vine near a karst wall. ZMKU R 01019 was collected from within a karst crevice and ZMKU R 01022 was found on a karst boulder. Two specimens (ZMKU R 01024 and ZMKU R 01026) were perched on concrete poles.

One individual (ZMKU R 01025) was found on a vine in a cave, approximately 10 m from the entrance, where the temperature was 26.1 °C and the relative humidity was 97.9%. *Cyrtodactyluswangkhramensis* sp. nov. appears to be sympatric with *Gehyramutilata* (Wiegmann, 1834).

##### Etymology.

The specific epithet *wangkhramensis* refers to the type locality at Tham Wangkhram in La-ngu District, Satun Province.

##### Comparison.

*Cyrtodactyluswangkhramensis* sp. nov. can be distinguished from other species in the *C.pulchellus* group by having a combination of weak tuberculation on the body; no tubercles on ventral surface of forelimbs, gular region or in ventrolateral body folds; 11–14 supralabial scales; 28–35 paravertebral tubercles; 19–21 longitudinal tubercle rows; 34–40 ventral scales; 32–36 femorprecloacal pores in males; deep precloacal groove in males; eleven dark caudal bands on original tail; light caudal bands on original tail infused with dark pigmentation in adults; and caudal tubercles 1/5–1/7 of anterior portion of tail. Additional comparisons between *Cyrtodactyluswangkhramensis* sp. nov. and other species in the *C.pulchellus* group are in Suppl. material 4.

*Cyrtodactyluswangkhramensis* sp. nov. is a member of Clade A which comprises *Cyrtodactylussungaiupe* sp. nov., *C.astrum*, *C.dayangbuntingensis*, *C.langkawiensis*, *C.lekaguli* and *C.stellatus*. It differs from those six species by uncorrected pairwise distances of ND2 of 6.59–11.30% (Table 2).

*Cyrtodactyluswangkhramensis* sp. nov. is sister to *Cyrtodactylussungaiupe* sp. nov. from which it differs by sequence divergence of 6.59–6.89% (Table 2) and strongly separated by PCA (Fig. 3). *Cyrtodactyluswangkhramensis* sp. nov. can be differentiated from its sister by having smaller maximum SVL of 98.8 mm (vs. 104.6 mm); caudal tubercles extending between 1/5 and 1/7 of anterior portion of tail (vs. 1/8–1/10 of the tail); and having significantly different mean values of mensural characters of AG_adj_, HW_adj_, EE_adj_ and EN_adj_ (*p* < 0.001–0.009; Table 5). It can be further separated from *Cyrtodactylussungaiupe* sp. nov. in having statistically significant different mean values of meristic characters of SL (*p* = 0.045; Table 5).

*Cyrtodactyluswangkhramensis* sp. nov. differs from *C.astrum* by having smaller maximum SVL of 98.8 mm (vs. 108.3 mm); 28–35 paravertebral tubercles (vs. 38–57); absence of scattered pattern of white tubercles on dorsum (vs. present); eleven dark caudal bands on the original tail (vs. 13 or 14); caudal tubercles extending between 1/5 and 1/7 of anterior portion of tail (vs. ≥ 1/3 of the tail); and having statistically significant different mean values of mensural characters of SVL, FL_adj_, TBL_adj_, HL_adj_, HW_adj_, HD_adj_, EE_adj_, ES_adj_, EN_adj_ and EL_adj_ (*p* < 0.001–0.004; Table 5). It can be further separated from *C.astrum* in having statistically significant different mean values of meristic characters of PVT and LRT (*p* < 0.001; Table 5).

*Cyrtodactyluswangkhramensis* sp. nov. can be separated from *C.dayangbuntingensis* by having 32–36 femoroprecloacal pores (vs. 26–29); dorsal band interspaces ratio (0.82–1.70 vs. 0.75); absence of scattered pattern of white tubercles on dorsum (vs. present); and one to two rows of postcloacal tubercles (vs. up to three rows).

*Cyrtodactyluswangkhramensis* sp. nov. differs from *C.langkawiensis* by having 32–36 femoroprecloacal pores (vs. 30); caudal tubercles extending between 1/5 and 1/7 of anterior portion of tail (vs. ≥ 1/3 of the tail); and having statistically significant different mean values of mensural characters of AG_adj_, HL_adj_, ES_adj_, EN_adj_ and IN_adj_ (*p* = 0.001–0.037; Table 5). Moreover, it can be differentiated from *C.langkawiensis* in having statistically significant different mean values of meristic characters of SL, IL, PVT, LRT and VS (*p* < 0.001–0.010; Table 5).

*Cyrtodactyluswangkhramensis* sp. nov. differs from *C.lekaguli* by having a smaller maximum SVL of 98.8 mm (vs. 108.5 mm); caudal tubercles extending between 1/5 and 1/7 of anterior portion of tail (vs. ≥ 1/3 of the tail); one to two rows of postcloacal tubercles (vs. one row) and having statistically significant different mean values of mensural characters of SVL, FL_adj_, HL_adj_, HW_adj_, HD_adj_, EE_adj_, ES_adj_, EN_adj_ and IO_adj_ (*p* < 0.001–0.007; Table 5). It can be differentiated from *C.lekaguli* in having statistically significant different mean values of meristic characters of SL, PVT and LRT (*p* = 0.001–0.036; Table 5).

*Cyrtodactyluswangkhramensis* sp. nov. differs from *C.stellatus* by having 32–36 femoroprecloacal pores (vs. 24–29); absence of precloacal pores in females (vs. present); absence of scattered pattern of white tubercles on dorsum (vs. present) and having statistically significant different mean values of mensural characters of AG_adj_, HL_adj_, HW_adj_ and HD_adj_ (*p* < 0.001–0.048; Table 5). It can be further separated from *C.stellatus* in having statistically significant different mean values of meristic characters of PVT (*p* < 0.001; Table 5).

## ﻿Discussion

This study discovered two unrecognised *Cyrtodactylus* species described here as *Cyrtodactylussungaiupe* sp. nov. and *Cyrtodactyluswangkhramensis* sp. nov. from karst areas in southern Thailand. It is noteworthy that *Cyrtodactylussungaiupe* sp. nov. and *Cyrtodactyluswangkhramensis* sp. nov. are closely related to insular sister species *C.dayangbuntingensis* and *C.langkawiensis* from the nearby offshore land-bridge Dayang Bunting and Langkawi Islands, respectively. The divergence of these sister clades (two new species and insular species) could have occurred during the Last Gracial Maximum when sea levels were lower than present-day exposing the land-mass connecting mainland and offshore islands (Sathiamurthy and Voris 2006). The higher post-glacial sea level of present day creates a geographic barrier separating these sister clades. Curiously, based on geographic proximity and habitat continuity, one would expect *Cyrtodactylussungaiupe* sp. nov. and *Cyrtodactyluswangkhramensis* sp. nov. to be more closely related to *C.stellatus* from Tarutao Island or *C.astrum* from the Thai-Malay border (Fig. 1). The short internodal branch lengths separating all the species of Clade A (Fig. 2) attests to the near simultaneous and somewhat stochastic divergence of these species from one another. This similar phylogeographic pattern is also found in other taxa, such as *Cnemaspis* in Southeast Asia (Grismer et al. 2014b; Wood et al. 2017; Ampai et al. 2022).

The two newly-recognised species, *Cyrtodactylussungaiupe* sp. nov. and *Cyrtodactyluswangkhramensis* sp. nov. resemble each other in morphology owing to their late and rapid divergence from one another and, thus, share a number of putative ancestral traits (e.g. Sheridan and Stuart [2018]). Moreover, they occur in the same karstic habitat where the same environmental pressures strongly influence their morphology and, thus, selection for change is presumably low (Grismer et al. 2020). Karsts are unique and usually occur as isolated landscapes which provided numerous suitable microhabitats that can be drivers of speciation (Grismer et al. 2021b) and, moreover, act as geographic barriers limiting the distributional range of organisms and, thus, usually bear high levels of endemism (Clements et al. 2006; Grismer et al. 2021b).

The numerous studies and explorations of unsurveyed areas, including offshore islands, mountain forests and karst outcrops and the use of integrative approaches in taxonomy have influenced the recent rise in gekkonid diversity in Thailand in the last decade (e.g. Ampai et al. (2022); Chomdej et al. (2022); Grismer et al. (2022a); Rujirawan et al. (2022); Yodthong et al. [2022]). This is strikingly clear for the *C.pulchellus* group. The two additional karst-associated species described here brings the number of species in the *C.pulchellus* species group from 17 to 19, of which six have been recognised in the Thai portion of these complexes’ distribution. Furthermore, it brings the number of *Cyrtodactylus* species in Thailand to 47 (Termprayoon et al. 2021a; Chomdej et al. 2022; Grismer et al. 2022a, 2023; Yodthong et al. 2022). The fact that the species diversity of the *C.pulchellus* group was underestimated by 19 fold as late as 2008 is a testament to the necessity of statistically and genetic-based integrative analyses. These analyses, coupled with descriptions of each species microhabitat will engender useful species-specific conservation management plans.

Currently, there are still numerous nearby karstic regions awaiting surveys which are needed to delimit the geographic ranges of any new and currently-recognised species. Further taxonomic studies using multi-locus genetic data are also needed to delimit species boundaries within other species groups throughout Thailand.

## Supplementary Material

XML Treatment for Cyrtodactylus sungaiupe

XML Treatment for Cyrtodactylus wangkhramensis

## ﻿Additional information

### Author ORCIDs

Korkhwan Termprayoon https://orcid.org/0000-0003-1903-3040

Attapol Rujirawan https://orcid.org/0000-0001-9179-6910

L. Lee Grismer https://orcid.org/0000-0001-8422-3698

Perry L. Wood Jr https://orcid.org/0000-0003-3767-5274

Anchalee Aowphol https://orcid.org/0000-0001-9504-4601

### Data availability

All of the data that support the findings of this study are available in the main text or supplementary information.

## ﻿Appendix I

Specimens examined.

*Cyrtodactylusastrum*: **Peninsular Malaysia**, Perlis, Gua Wang Burma: LSUHC 09928 (female) and LSUHC 100075 (male).

*Cyrtodactyluslekaguli*: **Thailand**, Trang Province, Na Yong District: 16 males, ZMKU R 00919–00921, ZMKU R 01027–01030, ZMKU R 01032–01034, THNHM 01781, THNHM 01784, THNHM 01787, THNHM 01791, FMNH 215987 (holotype), FMNH 176985, and 10 females, ZMKU R 00918, ZMKU R 01031, ZMKU R 01035–01036, THNHM 01694, THNHM 01777, THNHM 01788, THNHM 01790, FMNH 215985–215986 (paratypes).

*Cyrtodactylusstellatus*: **Thailand**, Satun Province, Tarutao Island: five males, ZMKU R 00905 (holotype), ZMKU R 00903, ZMKU R 00906–00907, ZMKU R 00915 (paratypes), and five females, ZMKU R 00899–00900, ZMKU R 00908–00909, ZMKU R 00913 (paratypes).

## Supplementary materials

Supplementary material 1 Specimen identification, localities, museum numbers and GenBank accession numbers Data type xlsx Explanation note The institutional abbreviations of examined specimens follow Sabaj (2020). https://binary.pensoft.net/file/905286This dataset is made available under the Open Database License (http://opendatacommons.org/licenses/odbl/1.0/). The Open Database License (ODbL) is a license agreement intended to allow users to freely share, modify, and use this Dataset while maintaining this same freedom for others, provided that the original source and author(s) are credited.Korkhwan Termprayoon, Attapol Rujirawan, L. Lee Grismer, Perry L. Wood Jr, Anchalee Aowphol

Supplementary material 2 Statistical comparisons of *Cyrtodactylussungaiupe* sp. nov., *Cyrtodactyluswangkhramensis* sp. nov. and closely-related species in Clade A of the *C.pulchellus* group Data type xlsx Explanation note Statistical comparisons of *Cyrtodactylussungaiupe* sp. nov., *Cyrtodactyluswangkhramensis* sp. nov. and closely-related species in Clade A of the *C.pulchellus* group from 14 size-adjusted morphometric and seven meristic characters. Data are given as mean, standard deviation (SD), ranges (min-max) and sample size (*N*) of each species. Statistical comparisons from ANOVA (*F*) and Kruskal-Wallis (*H*) test. Shaded cells denote significant p-values (< 0.05). Key: * tested by Welch’s *F* test. https://binary.pensoft.net/file/905287This dataset is made available under the Open Database License (http://opendatacommons.org/licenses/odbl/1.0/). The Open Database License (ODbL) is a license agreement intended to allow users to freely share, modify, and use this Dataset while maintaining this same freedom for others, provided that the original source and author(s) are credited.Korkhwan Termprayoon, Attapol Rujirawan, L. Lee Grismer, Perry L. Wood Jr, Anchalee Aowphol

Supplementary material 3 Descriptive morphometric (in millimetres) and meristic characters (min–max) of the type series Data type xlsx Explanation note Descriptive morphometric (in millimetres) and meristic characters (min–max) of the type series of *Cyrtodactylussungaiupe* sp. nov., *Cyrtodactyluswangkhramensis* sp. nov. and members of Clade A in the *Cyrtodactyluspulchellus* group. Abbreviations are defined in Materials and Methods. Key: Re = regenerated; L = left; R = right; / = data unavailable or inapplicable. https://binary.pensoft.net/file/905288This dataset is made available under the Open Database License (http://opendatacommons.org/licenses/odbl/1.0/). The Open Database License (ODbL) is a license agreement intended to allow users to freely share, modify, and use this Dataset while maintaining this same freedom for others, provided that the original source and author(s) are credited.Korkhwan Termprayoon, Attapol Rujirawan, L. Lee Grismer, Perry L. Wood Jr, Anchalee Aowphol

Supplementary material 4 Diagnostic characters of the *C.pulchellus* group including two new species Data type xlsx Explanation note Diagnostic characters of the *C.pulchellus* group including two new species, *Cyrtodactylussungaiupe* sp. nov. and *Cyrtodactyluswangkhramensis* sp. nov. Red shaded cells indicate the differences with *Cyrtodactylussungaiupe* sp. nov. Blue shaded cells indicate the differences with *Cyrtodactyluswangkhramensis* sp. nov. Yellow shaded cells indicate the differences with both *Cyrtodactylussungaiupe* sp. nov. and *Cyrtodactyluswangkhramensis* sp. nov. Keys: W = weak; P = prominent; / = data unavailable. Additional morphological data were obtained from the following literature (Grismer and Ahmad 2008; Grismer et al. 2012, 2014, 2016; Quah et al. 2019; Wood et al. 2020; Termprayoon et al. 2021a, 2021b). https://binary.pensoft.net/file/905289This dataset is made available under the Open Database License (http://opendatacommons.org/licenses/odbl/1.0/). The Open Database License (ODbL) is a license agreement intended to allow users to freely share, modify, and use this Dataset while maintaining this same freedom for others, provided that the original source and author(s) are credited.Korkhwan Termprayoon, Attapol Rujirawan, L. Lee Grismer, Perry L. Wood Jr, Anchalee Aowphol
